# Systematic Review of the Preclinical Technology Readiness of Orthopedic Gene Therapy and Outlook for Clinical Translation

**DOI:** 10.3389/fbioe.2021.626315

**Published:** 2021-03-17

**Authors:** Piers Wilkinson, Ilya Y. Bozo, Thomas Braxton, Peter Just, Elena Jones, Roman V. Deev, Peter V. Giannoudis, Georg A. Feichtinger

**Affiliations:** ^1^Division of Oral Biology, School of Dentistry, University of Leeds, Leeds, United Kingdom; ^2^CDT Tissue Engineering and Regenerative Medicine, Institute of Medical and Biological Engineering, University of Leeds, Leeds, United Kingdom; ^3^Federal Medical Biophysical Center, Federal Medical-Biological Agency of Russia, Moscow, Russia; ^4^Into Numbers Data Science GmbH, Vienna, Austria; ^5^Leeds Institute of Rheumatic and Musculoskeletal Medicine, University of Leeds, Leeds, United Kingdom; ^6^Ryazan State Medical University, Ryazan, Russia; ^7^Academic Department of Trauma and Orthopaedics, School of Medicine, University of Leeds, Leeds General Infirmary, Leeds, United Kingdom; ^8^NIHR Leeds Biomedical Research Centre, Chapel Allerton Hospital, Leeds, United Kingdom

**Keywords:** bone regeneration, gene therapy, preclinical models, translational medical research, orthopedic and trauma, regenerative medicine

## Abstract

Bone defects and improper healing of fractures are an increasing public health burden, and there is an unmet clinical need in their successful repair. Gene therapy has been proposed as a possible approach to improve or augment bone healing with the potential to provide true functional regeneration. While large numbers of studies have been performed *in vitro* or *in vivo* in small animal models that support the use of gene therapy for bone repair, these systems do not recapitulate several key features of a critical or complex fracture environment. Larger animal models are therefore a key step on the path to clinical translation of the technology. Herein, the current state of orthopedic gene therapy research in preclinical large animal models was investigated based on performed large animal studies. A summary and an outlook regarding current clinical studies in this sector are provided. It was found that the results found in the current research literature were generally positive but highly methodologically inconsistent, rendering a comparison difficult. Additionally, factors vital for translation have not been thoroughly addressed in these model systems, and the risk of bias was high in all reviewed publications. These limitations directly impact clinical translation of gene therapeutic approaches due to lack of comparability, inability to demonstrate non-inferiority or equivalence compared with current clinical standards, and lack of safety data. This review therefore aims to provide a current overview of ongoing preclinical and clinical work, potential bottlenecks in preclinical studies and for translation, and recommendations to overcome these to enable future deployment of this promising technology to the clinical setting.

## Introduction

**FIGURE 1:**
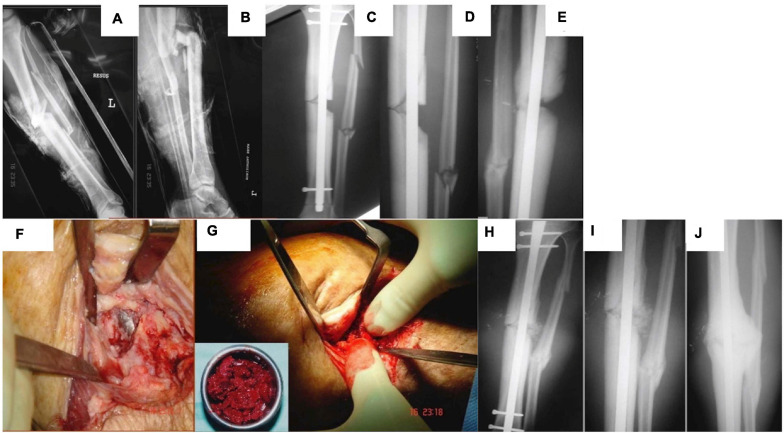
A male patient 29 years of age sustained a left tibial open fracture. **(A)** Initial anterior–posterior (AP). **(B)** Lateral radiographs of left tibia showing the midshaft fracture. **(C)** AP. **(D)** Lateral tibial radiographs showing that the fracture was stabilized with an intramedullary nail (note the tibial small defect laterally). **(E)** Lateral tibial radiograph at 4 months demonstrating lack of healing activity. **(F)** Intraoperative picture at 6 months showing non-union/bone loss and visualization of the nail. **(G)** Intraoperative picture showing bone grafting of the tibia with autologous bone graft. **(H)** AP postoperative. **(I)** Closer AP postoperative radiograph showing the area of small bone defect filled with the autologous bone graft. **(J)** AP radiograph 3 months lateral showing osseous healing of the tibial small defect.

While bone is highly competent at regeneration (Hadjidakis and Androulakis, 2006), a variety of situations can lead to damage that cannot be fully repaired by endogenous mechanisms. One of the most challenging examples is a major traumatic event resulting in significant bone loss, fragmentation, substantial damage to the surrounding soft tissue, or some combination of the above. Alternatively, other common etiologies for impaired healing and indications for augmentation of bone regeneration are surgical resection of a tumor or osteomyelitis (chronic bone infection), spinal fusion, and alveolar ridge atrophy caused by edentulism. Large bone defects that cannot be repaired by endogenous mechanisms leaving a permanent gap in the bone are termed “critical defects” (see Figure 1). Exactly how critical defects are defined is controversial, though sometimes a ratio of defect size to bone length is used. Typically, in humans, a defect of >1–2 cm in length where 50% of the bone circumference is lost will be critical (Lindsey et al., 2006; Spicer et al., 2012; Schemitsch, 2017). It is important to note that defect site and other factors have a major influence and may lead to defects that do not fit these parameters becoming critical or those that do fit them healing fully (Sanders et al., 2014; Schemitsch, 2017).

Complications in small fracture healing are commonly seen in older patients or those suffering with comorbidities, for example, disorders associated with systemic inflammation such as diabetes (Claes et al., 1999, 2012). This is typically manifested as delayed union, while cessation of fracture repair without full defect closure is often termed a “non-union” (Panagiotis, 2005; Tall, 2018). Non-unions are typically filled with fibrotic tissue and have some superficial similarities to a joint (see Figure 1), leading to the alternative name of pseudoarthrosis. With the increasing age of the general population and prevalence of chronic conditions (Anderson and Horvath, 2004; van Oostrom et al., 2016), the clinical burden of impaired fracture healing is likely to increase in the future. Consequently, there has been a great deal of interest in the use of regenerative medicine and tissue engineering to encourage impaired bone repair. Numerous combinations of genes, vectors, proteins, cells, scaffolds, and methods to apply them have been proposed or investigated (Grol and Lee, 2018).

When considering orthopedic bone repair, gene therapy has several advantages over competing methods. The present standard of regenerative, intraoperative care for such defects is a bone autograft. Bone is removed from a healthy donor site on the patient (typically the iliac crest or fibula) and then used to fill the original defect (see Figure 1) or space prefabricated with the Masquelet technique. While this approach now has a high success rate, it is a complex surgical procedure with a risk of donor site morbidity (Kuik et al., 2016). Alternative approaches include synthetic bone substitutes and allo- or xenografts; however, these are limited in osteoinductive potential (Buser et al., 2016; Wang and Yeung, 2017; Haugen et al., 2019; Sohn and Oh, 2019). Recombinant growth factors to encourage endogenous repair have seen use in the clinic; however, their effectiveness is impacted by short biological half-life, immunogenicity in some patients, and a host of other side effects (Talwar et al., 2001; Hwang et al., 2009). To ensure a physiologically relevant level of protein is present long enough to induce an effect of a supraphysiological initial dose (which may have adverse effects) (Aspenberg, 2013; James et al., 2016), repeat applications (clinically challenging), or a sustained release system (technically challenging) is required. Some attempts have been made to develop small molecules to encourage bone regeneration; however, these approaches face similar problems to those of growth factors (Paralkar et al., 2003; Laurencin et al., 2014). Gene therapy can potentially avoid all of these issues by delivering genetic blueprints. A single treatment or application gene therapy can lead to targeted, sustained, and controlled expression of therapeutic gene/s of interest, all of which can be tuned using vector and expression cassette design.

**FIGURE 2:**
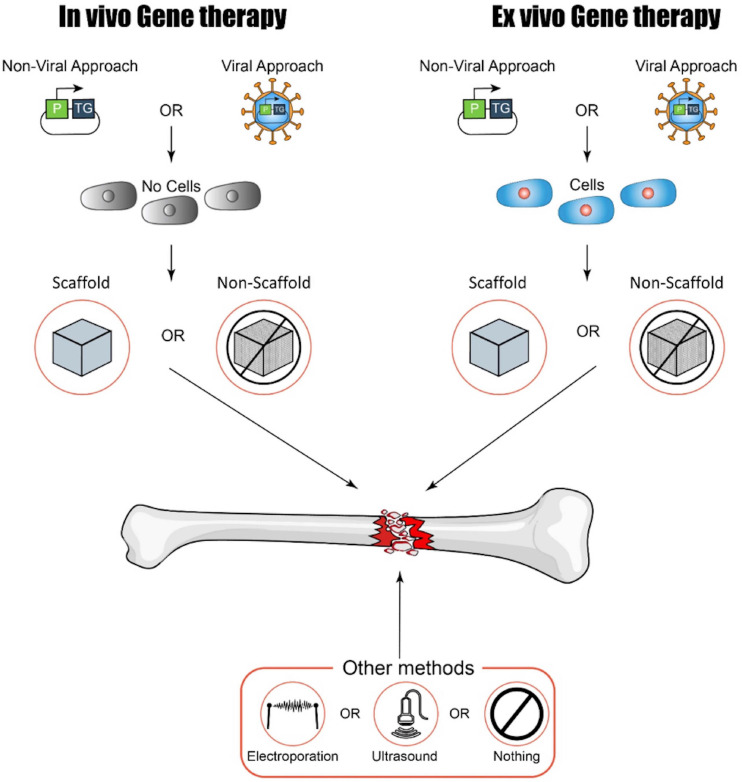
A summary of possible approaches to gene therapy for bone regeneration (kindly provided by D. Ilas). P, promoter; TG, transgene.

Various gene therapy approaches are now available and intensely studied for clinical translation. An important part of any gene therapy is the vector or method by which genes of interest are introduced to the target cells. Traditionally, a host of different viruses have been used for this purpose, making use of their natural adaptions for cell targeting and entry to deliver genetic information (Lundstrom, 2018). However, viral vectors can be cytotoxic (Büning and Schmidt, 2015) and have the potential to induce an immune response, possibly rendering the initial therapy or follow-up treatments ineffective (Nayak and Herzog, 2010). Furthermore, certain vectors encourage transgene integration in the host genome leading to potentially dangerous insertional mutagenesis effects (Knight et al., 2013; David and Doherty, 2017). Because of this, there is an increasing push toward non-viral gene therapies as a safer and therefore more easily translatable alternative in situations where only short-term transgene expression is required. In such methods, nucleic acids are introduced either “naked” or as part of a synthetic carrier such as a capsule or nanoparticle (Yin et al., 2014). While these methods are deemed to be often safer, the advantageous aspects of viral gene delivery are lost. Non-viral approaches typically show low efficiency and limited transgene persistence, and the host immune responses to both the carrier and its nucleic acid contents are still a concern in terms of safety and efficacy (Al-Dosari and Gao, 2009). A possible approach to mitigate the problems of both approaches is *ex vivo* gene therapy, a combination of cell and gene therapy (CGT). In such approaches, cells are removed from the patient or donor, transduced or transfected in the lab, screened for successful modification, and then reintroduced to the patient (Gregory-Evans et al., 2012) (see Figure 2). Such approaches are theoretically attractive and have already seen some success in humans in a variety of conditions (Kumar et al., 2016; Hirsch et al., 2017). However, they are expensive, time-consuming, and labor-intensive and introduce a host of new safety and regulatory issues. These limitations act as a major barrier to widespread clinical translation.

Another vital consideration is the therapeutic gene to be delivered. Many potential options have been proposed to encourage bone repair. Perhaps the most commonly used options to date are members of the bone morphogenetic protein (BMP) family of growth factors, due to their vital role in bone development, homeostasis, and repair (Bragdon et al., 2011; Wang et al., 2014). A popular alternative is vascular endothelial growth factor (VEGF) to encourage angiogenesis, vital in such a highly vascularized tissue as bone. These and numerous other options have been reviewed extensively elsewhere (Betz et al., 2018; Grol and Lee, 2018; Shapiro et al., 2018).

Each combination of delivery method, target cell population, and vector has a unique combination of positives and negatives, but common issues include targeting the correct cell population and establishing a suitable dose (Waehler et al., 2007; Yin et al., 2014). New approaches to vector and expression cassette design have been attempted to address these problems, but novel delivery methods may also be able to contribute to technological advancement and translatability. Scaffold or gene-activated matrix (GAM)-based delivery has drawn a great deal of interest from the tissue engineering community. In this approach, the gene therapy is immobilized on a 3D tissue engineering scaffold, allowing precise localization of the gene therapy to site of interest (Raisin et al., 2016; D’Mello et al., 2017). This technology has been developed further with the use of microbubble carriers, which can be sheared using ultrasound, providing unprecedented spatiotemporal control of gene delivery (Zhou et al., 2008; Nomikou et al., 2018). However, such approaches are still in their infancy and require further development.

Research and development of regenerative approaches for bone repair does not differ in terms of its iterative preclinical approach from other biomedical research fields; in fact, the large majority of preclinical animal research has been performed in small rodent models. While small rodent models have many advantages, they often fail to recapitulate key aspects of human biology (Chong et al., 2013; Seok et al., 2013; von Scheidt et al., 2017; King, 2018). Consequently, they can be poor predictors of the behavior of a treatment or therapy in humans. In the case of bone repair, the sheer difference in size, biomechanical loading, and biomechanics is particularly important, but mechanisms and rates of bone remodeling and biomechanics also vary widely between species (Reichert et al., 2009; Wancket, 2015; McGovern et al., 2018). Larger animal models are more similar to humans with regard to many of these factors, having the potential to be better models for orthopedic treatments. Rabbits (Inui et al., 1998; Murakami et al., 2002), sheep (Kirker-Head et al., 1998; den Boer et al., 2003), goats (Xu et al., 2005; Lian et al., 2009), dogs (Cook et al., 1994; Sumner et al., 2003), pigs (Lin et al., 2015; Bez et al., 2017), horses (Backstrom et al., 2004; Ishihara et al., 2008), and non-human primates (Andersson et al., 1978; Cook et al., 2002) have all been used as animal models for orthopedic implants or regenerative approaches to bone repair. Porcine bone has similar morphology and microstructure to that of humans; however, pigs are large and difficult to handle (Reichert et al., 2009; Wancket, 2015; Perleberg et al., 2018). Dogs also possess relevant bone physiology in several regards, but their status as a companion animal has prompted public concern over their use (O’Loughlin et al., 2008; McGovern et al., 2018). Sheep display favorable bone qualities, relative ease of handling, and lack of public objection to their use in research (Malhotra et al., 2014) but are still more expensive and difficult to house than are small rodents. There is no animal model that is unambiguously superior for bone research, and practical considerations such as animal handling, housing, and cost, which typically favor smaller models, cannot be ignored.

While preclinical animal experiments are required for the large majority of new drugs and treatments developed in Europe and the United States (Agency, 2008), regulators are currently highly flexible with regard to species choice. Industry guidelines published by the United States Food and Drug Administration (FDA) state that the species should demonstrate a biological response similar to that of humans and that the comparability of physiology and anatomy to humans should play a role (Center for Biologics Evaluation and Research, 2013). The European Medicines Agency (EMA) provides similar guidelines. It remains for the investigator to demonstrate that their species of choice is appropriate with regard to these and several additional factors (Agency, 2015). Testing in a second species is encouraged but sometimes not required, and there is no requirement to use larger animal models even in situations where they may be more representative of human biology.

## Methods

### Questions and Assessment

The PICO (Problem, Intervention, Comparison, and Outcome) question that the review attempts to answer is as follows: Has gene therapy been successfully applied to regenerate bone in large animal models? We also aimed to assess the various methodologies and approaches used across the field, making a thorough survey of methods that have been employed in large animal models. Finally, we assessed the risk of bias (RoB) of all of the publications using an established framework.

The information gathered to answer these questions was then used to conduct a technology readiness assessment (TRA). A TRA aims to estimate the technology readiness level (TRL) of a technology, in this case gene therapy for orthopedic bone regeneration. TRAs were first developed by the National Aeronautics and Space Administration (NASA) to allow consistent discussion of technical maturity across various technologies but have now become a popular assessment technique in many diverse fields. Simply, TRAs rank a technology on a 9- or 10-point scale of TRLs, with higher values indicating technological maturity. In bioscience/medicine, these scores correspond to closeness to clinical translation. TRAs are still uncommon in the fields of biomedicine and biotechnology, and consequently, there is no widely accepted framework for their use. In this case, a slightly modified version of the United States Department of Defense TRA Deskbook guidelines for biomedical TRLs (Office of the Director of Defense Research and Engineering, 2009) has been applied, a highly simplified version of which can be seen in Table 1. More detailed versions of the frameworks for biologics and medical devices are provided in Supplementary Table 4.

**TABLE 1:** A simplified biomedical technology readiness level (TRL) framework, based on the United States government DoD TRA Deskbook (Office of the director of defense and engineering, 2009).

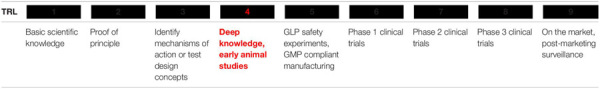

### Search Strategy

**FIGURE 3:**
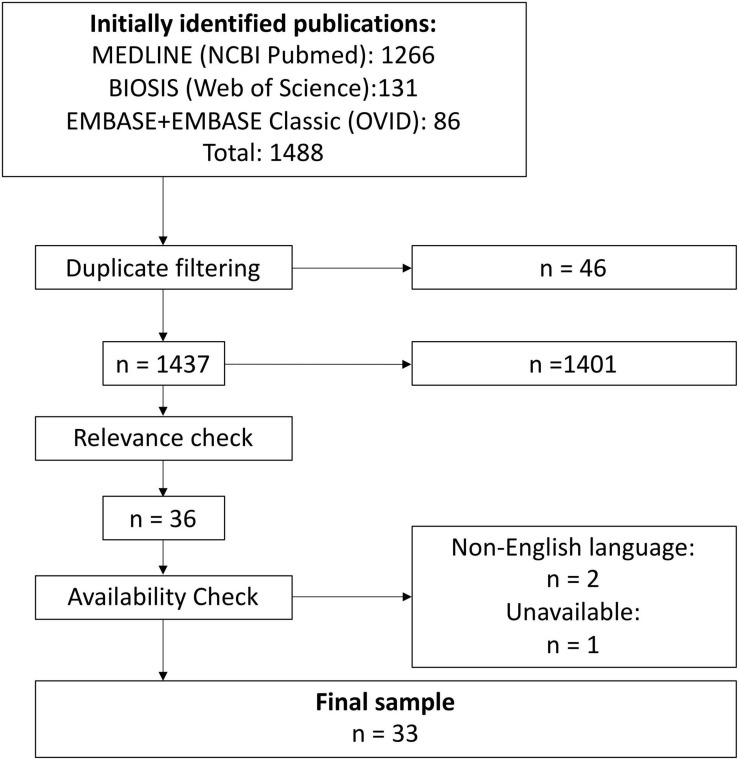
Overview of the preclinical literature search and filtering process.

The databases MEDLINE, EMBASE, and BIOSIS were searched in August 2019 for terms relating to animal models, gene therapy, and bone. Controlled or curated vocabulary was used for all databases, with an additional topic search added to the BIOSIS search due to a lack of sufficiently specific curated terms. The initial search returned 1,483 publications, which were then taken for filtering (see Figure 3).

See the Supplementary Information for a detailed description of the literature search.

### Filtering Strategy

References were exported from the databases into the reference management software EndNote X7. EndNote’s duplicate detection function and a manual search were used to filter for duplicates. A relevance search was then conducted based on the listed inclusion and exclusion criteria.

Inclusion criteria were as follows:

• Use of gene therapy,
• Focus on bone repair,
• Use of large animal models, and
• English language.

Exclusion criteria were as follows:

• Exclusive use of small animal models (rabbits and smaller);
• Exclusively *in vitro*; and
• Review articles, letters to the editor, and other non-research article types.

A first pass was made by searching the titles for terms associated with exclusion criteria (e.g., “mouse”). Identified titles were manually examined and excluded if a reasonable assumption that exclusion criteria were met could be made (e.g., the publication “Combination therapy with BMP-2 and a systemic RANKL inhibitor enhances bone healing in a mouse critical-sized femoral defect” was excluded at this stage, as there was no indication that a large animal model was used). The remaining publications were manually screened, with both the titles and abstracts consulted. Finally, an availability check was conducted. See Figure 3 for a summary of the search and filtering process.

### Automated Machine Reading and Visualizations

In order to provide a summary overview of the most common and recurring topics shared by the selected systematic review publications, we have developed a Python script, which automatically creates a visual representation of the most common key phrases, involved authors, journals, and publication years. We provide and maintain the current version as Jupyter Notebook under the following GitHub Repository: https://github.com/intonumbers/pubmed-insights.

First, we have queried the Entrez query and database system at the National Center for Biotechnology Information (NCBI) via their Entrez Programming Utilities (E-utilities) API for the corresponding 33 selected publications (Sayers, 2009).

To extract the most frequent key terms, we have included the values of the following keys per fetched publication dataset:

• Title,
• Abstract,
• Results,
• Keywords, and
• Conclusions.

Our data munging process’s first step was to remove all punctuation marks, special characters, and figures for each of the mentioned key–value pairs and replace all capital letters with their corresponding lowercase letters. We then removed common stop words (detailed stop-words list see GitHub repository) and lemmatized the remaining words using NLTK WordNet’s^1^ built-in Morphy function. Next, we have formed all *n*-grams between word-size 2 and 5 for all key–value pairs per publication dataset except for the keywords key–value pair, where we have defined each entry as *n*-gram regardless of their size.

Afterward, we have removed all *n*-gram duplicates per publication dataset.

For the final word cloud visualization, we have implemented a weighting score by multiplying the number of publications in which the *n*-gram occurs by the size of the corresponding *n*-gram and manually removed less meaningful *n*-grams (detailed ignore word list, see Supplementary Information). The program was applied to the dataset using the following parameters available in the graphical interface: Cloud Size = 100, Min Grams = 2, Max Grams = 5, Top Journals = 10, Long Gram Weight = ON, Remove incomplete author names = ON, and Remove Isolated Numbers = ON.

The size of each resulting word cloud item represents the magnitude of its *n*-gram’s score, taking into account the top 100 of the remaining entries. The size of each word cloud item represents the number of publications that the corresponding author name was found in the authors-key of the fetched publication datasets.

A bar chart was generated to depict the number of publications per year of the analyzed publication dataset. Another visualization was generated to show the distribution of the selected publications per journal. Only the top 10 journals based on the number of publications within the current dataset were considered.

### Manual Assessment of Publications

The following study characteristics were extracted from each publication: model species, number of animals, defect site/s, defect size and type, fixation method, inclusion and type of cells, inclusion and type of a scaffold/construct, vector, nature of therapy (i.e., *in vivo* or *ex vivo*), therapeutic gene/s, promoter of therapeutic gene/s, therapy dose (including carrier information, cell number, and modification efficiency, if available), therapy delivery site, time between creation of defect and administration of therapy, length of experiment, methods used to investigate bone regeneration, and the methods used to investigate immune response to, persistence of, and localization of gene therapy.

A RoB assessment was performed using the Systematic Review Centre for Laboratory animal Experimentation (SYRCLE) RoB tool for animal studies (Hooijmans et al., 2014). The tool is a modified version of the Cochrane group RoB tool, initially developed to standardize assessment of study biases in clinical randomized controlled trials (RCTs). The SYRCLE RoB tool has been modified to account for differences between clinical RCTs and preclinical animal experiments. Briefly, the tool provides a framework to assess if the study took adequate steps to avoid bias through randomization and investigator blinding at various stages, and other factors such as full data reporting. Relevant animal baseline characteristics were decided to be age and weight.

All publications were assessed independently by two reviewers (PW and TB) for both extracted study characteristics and the SYRCLE RoB tool. Results were then compared and discussed, with the consensus view presented here.

## Results

### Data Overview (Automated Analysis)

**FIGURE 4:**
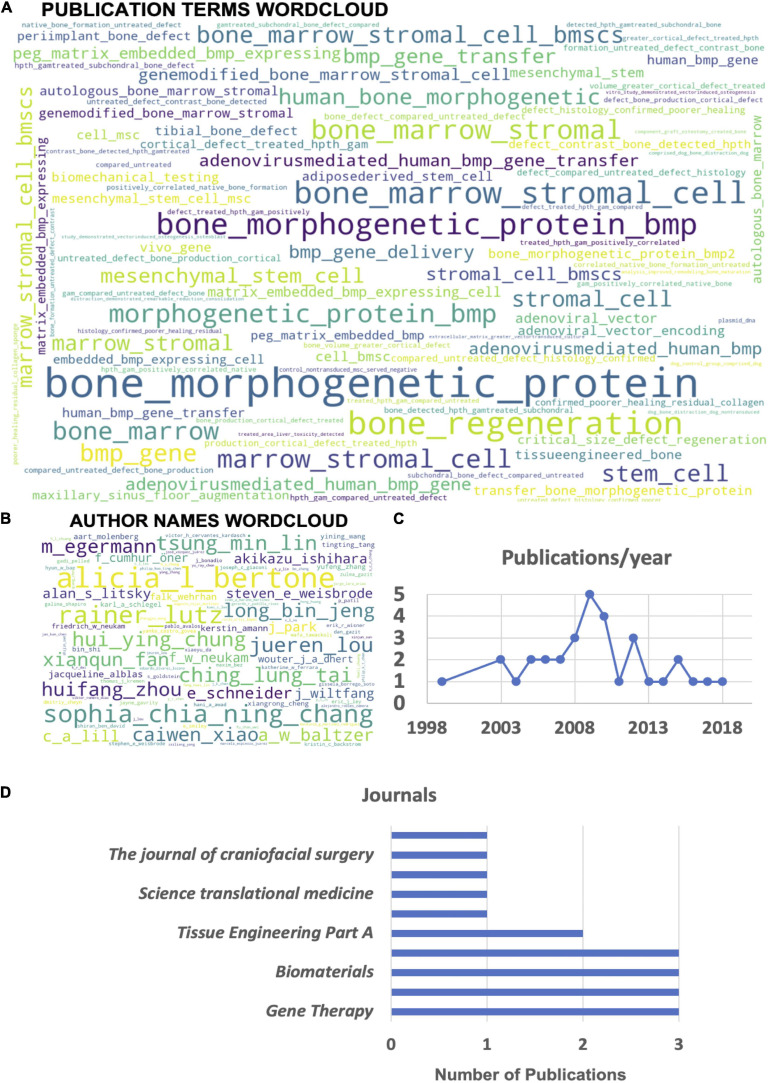
Automated machine reading analysis of the systematic review manuscript dataset (33 publications). **(A)** Word cloud depicting frequency of different *n*-grams across the abstracts of all selected publications. **(B)** Author name word cloud depicting the most common authors appearing on the highest frequency of publications within the analyzed dataset. **(C)** Overview of the yearly distribution of publication numbers within the dataset. **(D)** List of the top 10 journals with the most publications in the topic area within the analyzed dataset.

Automated machine reading data analysis of all 33 publication abstracts of the manuscripts selected for systematic review illustrates that the most common converging topic areas are centered around the use of bone marrow stromal cells (BMSCs) and BMP genes (Figure 4A). Furthermore, there is an indication of the relatively common use of adenoviral vectors for human BMP (hBMP) gene delivery in several studies. The author word cloud (Figure 4B) illustrates the most prolific authors found in the dataset but is not a representation of overall publication activity or leadership in the gene therapy field, as it might represent a skewed dataset in this regard due to the performed preselection. It gives, however, a suitable overview of the most active authors investigating orthopedic gene therapeutics in large animal models. Finally, publication activity in this field peaked around 2009 (Figure 4C) in the current dataset with a current stagnation since 2013, indicating a potential need for more intense preclinical research activity in this field in order to accelerate the translation of gene therapies for orthopedic indications. The most common journals identified in the current dataset (Figure 4D) are *Gene Therapy*, the *Journal of Orthopaedic Research*, and *Biomaterials*.

### Species and Models

**FIGURE 5:**
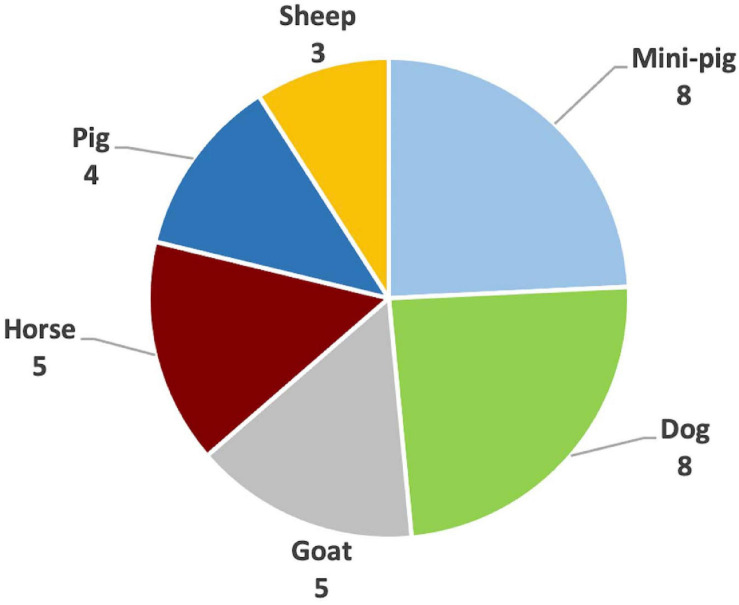
Summary of the species used in the reviewed publications.

Thirty-three publications were identified as relevant during the search. These publications were found to have used five different species. Dogs were used by eight publications (Bonadio et al., 1999; Zhang et al., 2007, 2009; Xiao et al., 2010; Castro-Govea et al., 2012; Deng et al., 2014; Liu et al., 2016; Kim et al., 2018), goats by five publications (Dai et al., 2005; Xu et al., 2005; Lian et al., 2009; Wegman et al., 2012; Loozen et al., 2015), sheep by three publications (Egermann et al., 2006a, b; Santoni et al., 2008), pigs by four publications (Park et al., 2007; Lutz et al., 2008; Wehrhan et al., 2012, 2013), mini-pigs by eight publications (Chang et al., 2003a, b, 2009, 2010; Chen et al., 2010; Kroczek et al., 2010; Lin et al., 2015; Bez et al., 2017), and horses by five publications (Backstrom et al., 2004; Ishihara et al., 2008, 2009, 2010; Southwood et al., 2012) (see Figure 5).

**FIGURE 6:**
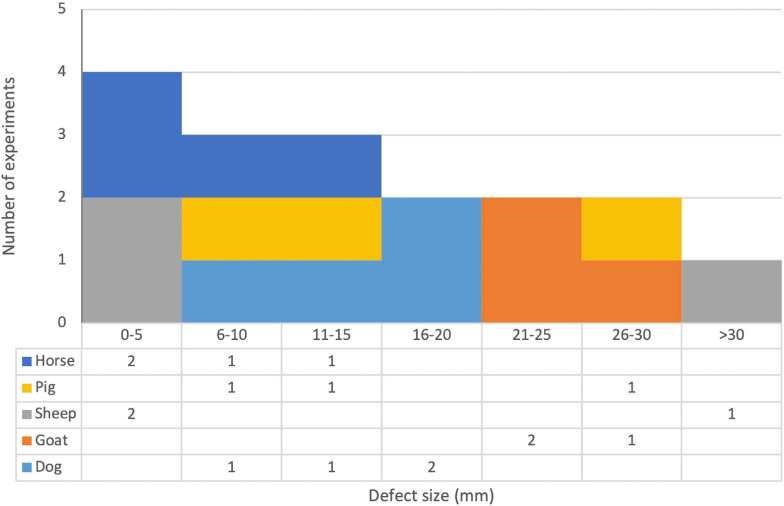
Stacked histogram and data table of defect sizes and species for publications that used full-thickness osteotomy defects in long bones. Note that publications that used multiple different defect sizes are included in all appropriate brackets and are counted twice if they used two different defect sizes that fell within the same bracket. See Table 3 for full details.

A wide variety of defect sites were investigated. Eight publications created defects in the maxillofacial region (not including the frontal bone). The specific sites investigated were the mandible (Zhang et al., 2007, 2009; Kroczek et al., 2010; Castro-Govea et al., 2012), inner orbit (Xiao et al., 2010; Deng et al., 2014), sinus (Liu et al., 2016), and infraorbital rim of the maxilla (Chang et al., 2003a). Seven publications created defects in the cranial or calvarial region (including the frontal bone) (Chang et al., 2003b, 2009, 2010; Park et al., 2007; Lutz et al., 2008; Wehrhan et al., 2012, 2013). Eleven publications created defects in the long bones of the limbs. The bones investigated were the tibia (Bonadio et al., 1999; Dai et al., 2005; Xu et al., 2005; Egermann et al., 2006a, b; Santoni et al., 2008; Bez et al., 2017), femur (Bonadio et al., 1999; Lian et al., 2009; Lin et al., 2015), ulna (Chen et al., 2010), and radius (Kim et al., 2018). Eight publications used a handful of other sites. These were the iliac crest (Egermann et al., 2006b; Ishihara et al., 2010; Loozen et al., 2015), various metacarpals and metatarsals (Backstrom et al., 2004; Ishihara et al., 2008; Ishihara et al., 2009; Southwood et al., 2012), ribs (Ishihara et al., 2010), and L1 vertebra (Wegman et al., 2012). Various different types of defect were created. Full-thickness osteotomies of long bones were made by 14 publications (Bonadio et al., 1999; Dai et al., 2005; Xu et al., 2005; Egermann et al., 2006a, b; Ishihara et al., 2008, 2009; Santoni et al., 2008; Lian et al., 2009; Chen et al., 2010; Southwood et al., 2012; Lin et al., 2015; Bez et al., 2017; Kim et al., 2018), with defect sizes ranging from 1 to 50 mm (see Figure 6). Drill holes were used by 11 publications (Bonadio et al., 1999; Backstrom et al., 2004; Egermann et al., 2006b; Park et al., 2007; Lutz et al., 2008; Chang et al., 2009; Ishihara et al., 2010; Wehrhan et al., 2012, 2013; Loozen et al., 2015). Cuboid and oval defects were used by six publications (Chang et al., 2003a, b, 2010; Lian et al., 2009; Zhang et al., 2009; Liu et al., 2016). A full-thickness osteotomy followed by distraction osteogenesis was used by two publications (Kroczek et al., 2010; Castro-Govea et al., 2012). One publication employed a decortication approach (Wegman et al., 2012).

In long-bone studies where fixation was potentially necessary, six used internal fixation methods; five used plates (Santoni et al., 2008; Southwood et al., 2012; Lin et al., 2015; Bez et al., 2017; Kim et al., 2018); one used intramedullary rods (Lian et al., 2009); and five used external approaches, with two using plates (Bonadio et al., 1999; Egermann et al., 2006a), two using circular/Ilizarov frames (Dai et al., 2005; Xu et al., 2005), and one using a custom approach (Egermann et al., 2006b). Three studies in long bones did not state if they used fixation; however, these studies all used sites where stability could be provided by other nearby bones (Ishihara et al., 2008, 2009; Chen et al., 2010).

**FIGURE 7:**
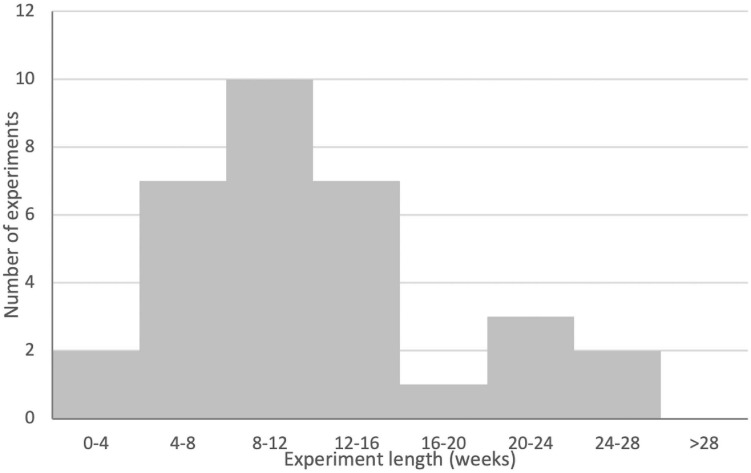
Histogram of experimental lengths for gene therapy groups across the publication set. Note that publications where experimental lengths were not clear or where different gene therapy treatment groups were sacrificed at different times are not included in this figure. Note for binning that experiments that landed on bin boundaries are included in the smaller bin (e.g., a 28-day experiment would be included in the 0- to 4-week bin). See Table 5 for full details.

All publications delivered their gene therapy directly into the defect. There was little variability in the length of time between the creation of the defect and the application of gene therapy. The large majority of publications applied their therapy immediately after defect creation, but five left several days between defect creation and therapy application. No chronic models were used. One publication applied their therapy 5 days post defect creation (Kroczek et al., 2010), while four applied their therapies 14 days post defect creation (Ishihara et al., 2008, 2009, 2010; Bez et al., 2017). The length of experiments (measured as the number of days between defect creation and sacrifice of the last experimental group) varied substantially, ranging from 28 (Park et al., 2007; Lutz et al., 2008) to 182 days (Dai et al., 2005) (Mean 93.2, SD 40.7; see Figure 7). The full results for species and model information are presented in Table 2.

**TABLE 2:** Study metrics 1.

Authors	Species	Total Number of animals	Defect site/s	Defect size (mm; where appropriate width × depth)	Fixation method
Backstrom et al., 2004	Horse	8	Metacarpal 3 (diaphysis and osteochondral)	6.5 × ? Drill hole	NA
Bez et al., 2017	Mini-pig (Yucatán)	18(?)	Tibia	10 full-thickness osteotomies	Internal, plate
Bonadio et al., 1999	Dog (mongrel)	Not stated	Femur, tibia	8 × 8 drill hole or 20,16,10 full width	External, plate
Castro-Govea et al., 2012	Dog (mongrel)	9	Mandible	Distraction osteogenesis: 1 mm/day for 10 days	Distraction device
Chang et al., 2003a	Mini-pig (Mitsae)	20	Maxilla (infraorbital rim)	30 × 12 rectangular (full thickness)	NA
Chang et al., 2010	Mini-pig (Mitsae)	40	Cranium (bilateral, bone unspecified)	20 × 50 oval (full thickness)	NA
Chang et al., 2003b	Mini-pig (Mitsae)	20	Cranium (bilateral, bone unspecified)	20 × 50 oval (full thickness)	NA
Chang et al., 2009	Mini-pig (Mitsae)	22	“Skull” (bone unspecified)	40 × unspecified (full thickness) drill hole	NA
Chen et al., 2010	Mini-pig (Guizhou)	18	Ulna	15 (?)	None stated (none?)
Dai et al., 2005	Goat (?)	26	Tibia	26 full-thickness osteotomies	External, circular
Deng et al., 2014	Dog (beagle)	18	Orbit (bilateral, various bones)	12 × unspecified (full thickness)	NA
Egermann et al., 2006a	Sheep (white mountain)	12	Tibia	3 full-thickness osteotomies	External, plate
Egermann et al., 2006b	Sheep (white mountain)	28	Iliac crest, tibia	Iliac crest: 20 × 5 drill hole; tibia: 3	External, custom
Ishihara et al., 2008	Horse	12	Fourth metatarsal, second metatarsal	Mt4: 1, Mt2: 10	Not stated (none?)
Ishihara et al., 2009	Horse	6	Fourth metacarpal and metatarsal	1 full-thickness osteotomy	Not stated (none?)
Ishihara et al., 2010	Horse	6	Ribs 10 or 11 (3 defects/rib, 6 defects/animal), ilium (4 defects/animal)	8 × 10 drill holes	NA
Kim et al., 2018	Dog (beagle)	12	Radius	15 full-thickness osteotomies	Internal, plate
Kroczek et al., 2010	Mini-pig (Göttingen)	24	Mandible	Distraction osteogenesis: 1.5 mm/day for 10 days	Distraction device
Lin et al., 2015	Mini-pig (Lee-Sung)	9	Femur	30 full-thickness osteotomies	Internal, plate
Liu et al., 2016	Dog (beagle)	6	Sinus (bilateral)	10 × 15 rectangular (full thickness)	NA
Loozen et al., 2015	Goat (breed?)	10	Iliac crest (bilateral, 2 per side)	6.4 × 10 drill hole	NA
Lutz et al., 2008	Pig (?)	8	Calvarium (bone unspecified, 9 defects per animal)	10 × 7 drill holes	NA
Park et al., 2007	Pig (?)	8	Frontal bone (9 defects/animal)	10 × 7 drill holes	NA
Santoni et al., 2008	Sheep (Rambouillet × Columbia)	24	Tibia	50 full-thickness osteotomies	Internal, plate
Southwood et al., 2012	Horse	15	Metacarpal 4 (bilateral)	15 full-thickness osteotomies	Internal, plate
Wegman et al., 2012	Goat (Dutch milk)	4	L1 vertebra or muscle pockets adjacent to spine	Unspecified size decortication	Other?
Wehrhan et al., 2013	Pig (?)	20	“Frontal skull” (9 defects/animal)	10 × 10 drill hole	NA
Wehrhan et al., 2012	Pig (?)	15	Calvarium (bone unspecified, 9 defect/animal)	10 × 10 drill hole	NA
Xiao et al., 2010	Dog (beagle)	16	Orbit (bilateral, various bones)	12 × unspecified (full thickness)	NA
Xu et al., 2005	Goat (?)	19	Tibia	21 full-thickness osteotomies	External, round
Zhang et al., 2009	Dog (mongrel)	6	Mandible	6 × 4 × 5 cuboid defect	NA
Zhang et al., 2007	Dog (mongrel)	9	Mandible	6 × 4 × 5 cuboid defect	NA
Lian et al., 2009	Goat	20	Femur	25 full-thickness osteotomies	Internal, intramedullary rod

*Question marks indicate where authors did not clearly provide the following information: species—animal breed; total number of animals—total number of animals is not clearly stated, and therefore, values are estimates from provided group sizes; defect size—one or more dimensions of the defect were not clearly provided.*

### Scaffolds and Biomaterials

**FIGURE 8:**
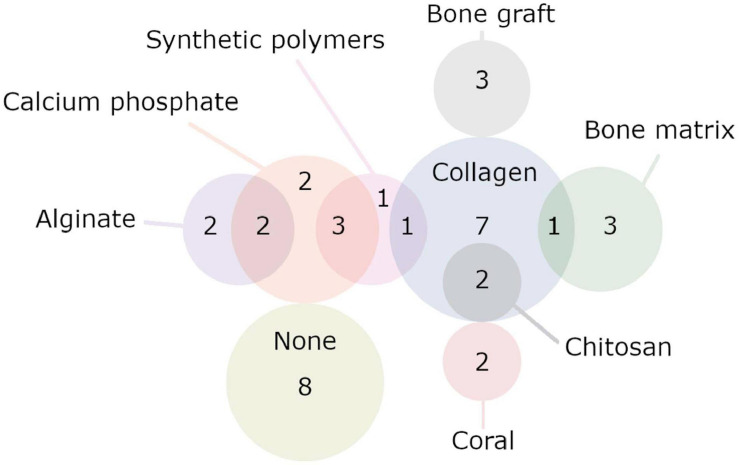
Set diagram representing the various biomaterials used by the publications. Here, only publications that used a single biomaterial or a combination of two are displayed. Two publications that used more complicated scaffolds/constructs made up of three or more materials are not included here. Note that publications were counted multiple times if they used multiple different experimental groups with different biomaterials. Publications included in the “None” group never used a biomaterial; it does not include publications that used a biomaterial but included a no-biomaterial group. See Table 4 for full details.

Twenty-five publications used some form of scaffold or construct to fill the defect they had created, with a wide variety of scaffolds being employed (Figure 8) (Bonadio et al., 1999; Chang et al., 2003a, b, 2009, 2010; Backstrom et al., 2004; Dai et al., 2005; Park et al., 2007; Zhang et al., 2007, 2009; Lutz et al., 2008; Santoni et al., 2008; Lian et al., 2009; Chen et al., 2010; Xiao et al., 2010; Castro-Govea et al., 2012; Wegman et al., 2012; Wehrhan et al., 2012, 2013; Deng et al., 2014; Lin et al., 2015; Loozen et al., 2015; Liu et al., 2016; Bez et al., 2017; Kim et al., 2018). Ten publications used scaffolds that contained multiple substances or were multiphasic (Chang et al., 2003a, 2009; Dai et al., 2005; Zhang et al., 2007, 2009; Wegman et al., 2012; Wehrhan et al., 2012, 2013; Loozen et al., 2015; Kim et al., 2018). Seven publications used scaffolds that were entirely composed of collagen (Bonadio et al., 1999; Chang et al., 2003b, 2010; Backstrom et al., 2004; Park et al., 2007; Lutz et al., 2008; Bez et al., 2017), and four publications used scaffolds that contained it in combination with other materials (Chang et al., 2003a; Dai et al., 2005; Zhang et al., 2007, 2009). One publication used tricalcium phosphate (TCP) alone (Deng et al., 2014), and five used some form of calcium phosphate in combination with other materials (Wegman et al., 2012; Wehrhan et al., 2012, 2013; Loozen et al., 2015; Kim et al., 2018). Two publications used a scaffold solely composed of treated bone matrix (Castro-Govea et al., 2012; Liu et al., 2016), and three used bone matrix in combination with other materials (Dai et al., 2005; Chen et al., 2010; Kim et al., 2018). Two publications employed coral scaffolds (Lian et al., 2009; Xiao et al., 2010), and none used coral in combination with another material. Alginate was used by three publications, two investigated alginate only scaffolds (Chang et al., 2010; Wegman et al., 2012), and two investigated it in combination with other materials (Wegman et al., 2012; Loozen et al., 2015) (note that Wegman et al., 2012 tried both). Chitosan was employed by two publications, both times in combination with collagen (Zhang et al., 2007, 2009). One publication used gelatin in combination with various other materials in a complex multiphasic scaffold (Chang et al., 2009). A variety of biodegradable polymers were used by a small number of publications, largely in combination with other materials. Poly(ε-caprolactone) (PCL) (Kim et al., 2018), poly(L-lactide acid) (PLLA) (Chang et al., 2003a), and poly(ethylene glycol) (PEG) (Wehrhan et al., 2012, 2013) were used with other materials, while one publication used poly(lactic-*co*-glycolic acid) (PLGA)-only scaffold (Lin et al., 2015). Two studies used autologous bone grafts (Park et al., 2007; Lutz et al., 2008). Eight publications did not use a scaffold or construct (Xu et al., 2005; Egermann et al., 2006a, b; Ishihara et al., 2008, 2009, 2010; Kroczek et al., 2010; Southwood et al., 2012). See Table 3 for full details.

**TABLE 3:** Study metrics 2.

Authors	Cells	Scaffold/construct	Vector system	*In vivo* or *ex vivo* gene therapy?
Backstrom et al., 2004	No	Collagen sponge	Plasmid	*In vivo*
Bez et al., 2017	No	Collagen	Plasmid + microbubbles	*In vivo*
Bonadio et al., 1999	No	Collagen (mixed with the DNA)	Plasmid	*In vivo*
Castro-Govea et al., 2012	Autologous BMSCs	Demineralized human bone matrix	Adenovirus 5	*Ex vivo*
Chang et al., 2003a	Autologous BMSCs	PLLA + collagen	Adenovirus 5	*Ex vivo*
Chang et al., 2010	Autologous BMSCs	Alginate or collagen gel	Adenovirus 5	*Ex vivo*
Chang et al., 2003b	Autologous BMSCs	Collagen	Adenovirus 5	*Ex vivo*
Chang et al., 2009	Autologous (?) BMSCs	Multiphasic: layers of pluronic F127 gel + gelatin/TCP ceramic/glutaraldehyde composite	Adenovirus 5	*Ex vivo*
Chen et al., 2010	Autologous (?) ADSCs	Acellular bone matrix	Plasmid	*Ex vivo*
Dai et al., 2005	Autologous BMSC	Biphasic calcined bone + collagen	Adenovirus 5	*Ex vivo*
Deng et al., 2014	Autologous BMSCs	β-TCP	Adenovirus (?)	*Ex vivo*
Egermann et al., 2006a	No	No	Adenovirus 5	*In vivo*
Egermann et al., 2006b	No	No	Adenovirus 5	*In vivo*
Ishihara et al., 2008	No	No	Adenovirus 5	*In vivo*
Ishihara et al., 2009	Autologous fibroblasts	No	Adenovirus (?)	*Ex vivo*
Ishihara et al., 2010	Autologous dermal Fbs	No	Adenovirus 5	*Ex vivo*
Kim et al., 2018	Allogeneic ADSCs	PCL/β-TCP, additional demineralized bone matrix (DBM) particles for one group	Lentivirus	*Ex vivo*
Kroczek et al., 2010	No	No	Plasmid + liposome	*In vivo*
Lin et al., 2015	Allogeneic ADSCs	PLGA	Baculovirus	*Ex vivo*
Liu et al., 2016	Autologous BMSCs	Bio-Oss (deproteinized bovine bone matrix)	Lentivirus	*Ex vivo*
Loozen et al., 2015	Allogeneic BMSC or None	BCP + alginate	Plasmid	*In vivo*
Lutz et al., 2008	No	Collagen or autologous bone graft	Plasmid + liposome	*In vivo*
Park et al., 2007	No	Collagen sponge or autologous bone graft	Plasmid + liposome	*In vivo*
Santoni et al., 2008	No	Allograft	Plasmid + PLGA microspheres	*In vivo*
Southwood et al., 2012	No	No	Adenovirus 5	*In vivo*
Wegman et al., 2012	Allogeneic BMSC or none	Biphasic calcium phosphate + alginate, or only alginate	Plasmid	*Hybrid*
Wehrhan et al., 2013	Human fetal osteoblasts	PEG hydrogel matrix or PEG membrane, with HA/TCP	Plasmid	*Ex vivo*
Wehrhan et al., 2012	Human fetal osteoblasts	PEG hydrogel + biphasic HA/TCP	Plasmid	*Ex vivo*
Xiao et al., 2010	Autologous BMSCs	Biocoral	Adenovirus (?)	*Ex vivo*
Xu et al., 2005	Autologous BMSCs	No	Adenovirus 5	*Ex vivo*
Zhang et al., 2009	No	Chitosan + collagen	Adenovirus (?)	*In vivo*
Zhang et al., 2007	No	Chitosan + collagen	Adenovirus (?)	*In vivo*
Lian et al., 2009	Autologous (?) BMSC	Coral	Adenovirus (?)	*Ex vivo*

*Question marks indicate where information was unclear. Cells—donor and recipient animals are not clearly identified, and therefore, cell status is inferred from the text; Vector system—virus serotype not provided.*

*BMSC, bone marrow stromal cell; ADSC, adipose-derived mesenchymal stem cell; PLGA, poly(lactic-co-glycolic acid); PEG, poly(ethylene glycol); HA, hydroxyapatite; TCP, tricalcium phosphate.*

### Vector Systems, Therapeutic Genes, Treatment Approaches, and Dosing

**FIGURE 9:**
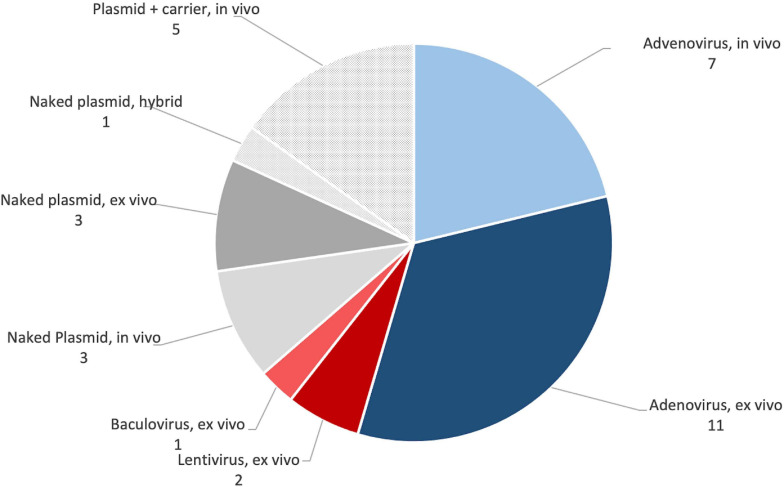
Summary of the popularity of different gene transfer approaches in preclinical studies. See Table 5 for full details.

The vector systems used were less variable (see Figure 9). Approximately two thirds of the publications used viral vectors, accounting for 21 of the 33 publications in the set. Eighteen publications used adenovirus, with 12 clearly indicating that they used serotype 5 (Chang et al., 2003a, b, 2009, 2010; Dai et al., 2005; Xu et al., 2005; Egermann et al., 2006a, b; Ishihara et al., 2008, 2010; Castro-Govea et al., 2012; Southwood et al., 2012). The remaining six did not clearly state which form of adenovirus they used (Zhang et al., 2007, 2009; Ishihara et al., 2009; Lian et al., 2009; Xiao et al., 2010; Deng et al., 2014). Two publications used lentivirus (Liu et al., 2016; Kim et al., 2018), and a single publication used baculovirus (Lin et al., 2015). The remaining 12 publications used non-viral gene delivery methods. Seven used “naked” plasmids (Bonadio et al., 1999; Backstrom et al., 2004; Chen et al., 2010; Wegman et al., 2012; Wehrhan et al., 2012, 2013; Loozen et al., 2015), and five used plasmids with some form of carrier (Park et al., 2007; Lutz et al., 2008; Santoni et al., 2008; Kroczek et al., 2010; Bez et al., 2017). Eighteen publications used *ex vivo* approaches (Chang et al., 2003a, b, 2009, 2010; Dai et al., 2005; Xu et al., 2005; Ishihara et al., 2009, 2010; Lian et al., 2009; Chen et al., 2010; Xiao et al., 2010; Castro-Govea et al., 2012; Wehrhan et al., 2012, 2013; Deng et al., 2014; Lin et al., 2015; Liu et al., 2016; Kim et al., 2018), 14 used *in vivo* approaches (Bonadio et al., 1999; Backstrom et al., 2004; Egermann et al., 2006a, b; Park et al., 2007; Zhang et al., 2007, 2009; Ishihara et al., 2008; Lutz et al., 2008; Santoni et al., 2008; Kroczek et al., 2010; Southwood et al., 2012; Loozen et al., 2015; Bez et al., 2017), and one used a hybrid approach, where cells were sometimes included in a gene-activated scaffold (Wegman et al., 2012). See Table 3 for full details.

Therapeutic genes were similarly consistent. Twenty-five publications used BMP-2 (Chang et al., 2003a, b, 2009, 2010; Dai et al., 2005; Xu et al., 2005; Egermann et al., 2006a, b; Park et al., 2007; Ishihara et al., 2008, 2009, 2010; Lutz et al., 2008; Santoni et al., 2008; Chen et al., 2010; Kroczek et al., 2010; Xiao et al., 2010; Castro-Govea et al., 2012; Southwood et al., 2012; Wegman et al., 2012; Wehrhan et al., 2012, 2013; Deng et al., 2014; Lin et al., 2015; Loozen et al., 2015), in three cases in combination with another gene (Chen et al., 2010; Southwood et al., 2012; Deng et al., 2014). Of these, 19 used hBMP-2 (Chang et al., 2003a, b, 2009, 2010; Dai et al., 2005; Xu et al., 2005; Egermann et al., 2006a, b; Park et al., 2007; Ishihara et al., 2008; Lutz et al., 2008; Santoni et al., 2008; Chen et al., 2010; Xiao et al., 2010; Castro-Govea et al., 2012; Southwood et al., 2012; Deng et al., 2014; Lin et al., 2015; Loozen et al., 2015), with six not indicating the species of origin of their gene (Ishihara et al., 2009, 2010; Kroczek et al., 2010; Wegman et al., 2012; Wehrhan et al., 2012, 2013). Five publications used BMP-7, with three using hBMP-7 (Zhang et al., 2007; Lian et al., 2009; Southwood et al., 2012), one using canine BMP-7 (Kim et al., 2018) (in a canine defect model, the only clearly indicated case where the model species and the species of origin of the therapeutic gene matched) and one not indicating the species of origin (Zhang et al., 2009). Three publications used VEGF (Chen et al., 2010; Deng et al., 2014; Lin et al., 2015), in all cases human. In all cases, VEGF was used either in comparison with or simultaneously to BMP-2. Two publications used parathyroid hormone (PTH), in both cases human and not in combination with other genes (Bonadio et al., 1999; Backstrom et al., 2004). BMP-6 was used by two publications (Ishihara et al., 2008; Bez et al., 2017), in both cases human and used in isolation, although compared with BMP-2 in one case. Individual publications used DMP1 (Liu et al., 2016) (alone) and PDGF-B (Zhang et al., 2009) (with or without BMP-7). In both cases, the species of origin of the genes was not indicated. See Table 4 for full details of the therapeutic genes used.

**TABLE 4:** Study metrics 3.

Authors	Therapeutic gene/s	Promoter/s	Dose	Delivery site	Length of time to gene therapy application after creation of defect (days)	Longest follow-up period after defect creation (days)
Backstrom et al., 2004	hPTH	Not stated	100 mg	Defect	0	84–91 (“during week 13”)
Bez et al., 2017	hBMP-6	CMV	1 mg DNA, 1 × 10^7^ microbubbles	Defect	14	56
Bonadio et al., 1999	hPTH	CMV-IE	1 to 100 mg DNA	Defect	0	Various (?)
Castro-Govea et al., 2012	hBMP-2	CMV	Unknown cells/scaffold (MOI ∼ 80, no transduction efficiency)	Defect	0	70
Chang et al., 2003a	hBMP-2	CMV	1.5 × 10^7^ cells (MOI 50, no transduction efficiency)	Defect	0	∼90 (3 months)
Chang et al., 2010	hBMP-2	CMV	5 × 10^7^ cells/scaffold (no MOI, no transduction efficiency)	Defect	0	∼90 (3 months)
Chang et al., 2003b	hBMP-2	CMV	3 × 10^8^ cells/defect (No MOI or transduction efficiency)	Defect	0	∼90 (3 months)
Chang et al., 2009	hBMP-2	CMV	3 × 10^8^ cells/defect (MOI 50)	Defect	0	∼180 (6 months)
Chen et al., 2010	hBMP-2 and hVEGF	Not stated	Unknown cells/scaffold (no transfection efficiency)	Defect	0 (?)	84
Dai et al., 2005	hBMP-2 (? Inconsistently labeled)	CMV	1 × 10^8^ cells (MOI 200, no transduction efficiency)	Defect	0	182
Deng et al., 2014	hBMP-2 and/or hVEGF	CMV	1 × 10^7^ cells/scaffold (MOI 150, 90% efficiency with separate GFP reporter virus)	Defect	0	112
Egermann et al., 2006a	hBMP-2	CMV	1 × 10^11^ particles	Defect	0	56
Egermann et al., 2006b	hBMP-2	CMV (-IE?)	1 × 10^11^ particles	Defect	0	56
Ishihara et al., 2008	hBMP-2 or hBMP-6	CMV	5 × 10^11^ particles	Defect	14	56 (42 post therapy)
Ishihara et al., 2009	BMP-2 (?)	Not stated	5 × 10^7^ cells (MOI 200, >95% efficiency with separate GFP reporter virus)	Defect	14	56 (42 post therapy)
Ishihara et al., 2010	BMP-2 (?)	CMV	5 × 10^7^ cells (MOI 200, >80% efficiency with separate GFP reporter virus)	Defect	14	56 (42 post therapy)
Kim et al., 2018	Dog BMP-7	EF-1α	Unknown cells/scaffold (no transduction efficiency, but selected with antibiotic resistance)	Defect	0	84
Kroczek et al., 2010	BMP-2 (?)	CMV	25 μg DNA + Liposome (?)	Defect	5 (?)	43 (?)
Lin et al., 2015	hBMP-2 or hVEGF	CMV	1 × 10^8^ cells (MOI 150, no transduction efficiency)	Defect	0	84 (?)
Liu et al., 2016	DMP1 (?)	Not stated	Unknown cells/scaffold (MOI 4, 95% transduction efficiency at day 3 post transduction as established by fluorescence microscopy for GFP marker gene)	Defect	0	84
Loozen et al., 2015	hBMP-2	CMV	Unknown cells/scaffold (no transfection efficiency)	Defect	0 (?)	84
Lutz et al., 2008	hBMP-2	CMV	12 μg DNA/defect	Defect	0	28
Park et al., 2007	hBMP-2	CMV	12 μg DNA	Defect	0	28
Santoni et al., 2008	hBMP-2	Not stated	100 mg microbubble + plasmid mix (?)	Defect	0	∼120 (4 months)
Southwood et al., 2012	hBMP-2 and hBMP-7	CMV	2 × 10^11^ particles	Defect	0	112
Wegman et al., 2012	His-BMP-2 (?)	Not stated	3 μg DNA, 3 × 10^6^ cells (no transfection efficiency)	Defect	0	112
Wehrhan et al., 2013	BMP-2 (?)	CMV	1.5 × 10^6^ cells (no MOI or transfection efficiency)	Defect	0	84
Wehrhan et al., 2012	BMP-2 (?)	CMV	1.5 × 10^6^ cells (no MOI or transfection efficiency)	Defect	0	84
Xiao et al., 2010	hBMP-2	Not stated	1 × 10^7^ cells/scaffold (MOI 150, no transduction efficiency)	Defect	0	168
Xu et al., 2005	hBMP-2	CMV	3 × 10^8^ cells (MOI 200, transduction efficiency)	Defect	0	168
Zhang et al., 2009	BMP-7 (?) and/or PDGF-B (?)	Not stated	1 × 10^6^ cells/scaffold (no MOI, no transduction efficiency)	Defect	0	84
Zhang et al., 2007	hBMP-7	CMV	1 × 10^6^ cells/scaffold (no MOI, no transduction efficiency)	Defect	0	84
Lian et al., 2009	hBMP-7	Not stated	5 × 10^7^ cells (MOI 100, no transduction efficiency)	Defect	0	Experimental: ∼150 (5 months), Control: ∼240 (8 months)

*In the “Therapeutic gene/s” column, an “h” prefix to a gene name indicates the gene is of human origin. Question marks indicate where the following information was unclear. Cells—donor and recipient animals are not clearly identified therefore cell source is inferred from the text; Vector system—virus serotype not provided; Longest follow-up period—Length not clearly indicated in the text and therefore inferred from figures.*

*PTH, parathyroid hormone; BMP, bone morphogenetic protein; CMV, cytomegalovirus; CMV-IE, cytomegalovirus immediate-early; MOI, multiplicity of infection; VEGF, vascular endothelial growth factor; GFP, green fluorescent protein.*

Promoter choice for expression cassettes was highly consistent. Twenty-three publications used cytomegalovirus (CMV) promoter or variants such as immediate-early (CMV-IE) (Bonadio et al., 1999; Chang et al., 2003a, b, 2009, 2010; Dai et al., 2005; Xu et al., 2005; Egermann et al., 2006a, b; Park et al., 2007; Zhang et al., 2007; Ishihara et al., 2008, 2010; Lutz et al., 2008; Kroczek et al., 2010; Castro-Govea et al., 2012; Southwood et al., 2012; Wehrhan et al., 2012, 2013; Deng et al., 2014; Lin et al., 2015; Loozen et al., 2015; Bez et al., 2017). One publication used the human elongation factor-1 alpha (EF-1α) promoter (Kim et al., 2018). Nine publications did not clearly state which promoter was used (Backstrom et al., 2004; Santoni et al., 2008; Ishihara et al., 2009; Lian et al., 2009; Zhang et al., 2009; Chen et al., 2010; Xiao et al., 2010; Wegman et al., 2012; Liu et al., 2016). See Table 4 for full details.

Twenty publications used cells as part of their therapy. Autologous BMSCs were overwhelmingly the most popular option, being used by 11 publications (Chang et al., 2003a, b, 2009, 2010; Dai et al., 2005; Xu et al., 2005; Lian et al., 2009; Xiao et al., 2010; Castro-Govea et al., 2012; Deng et al., 2014; Liu et al., 2016). Various other cell types were used by two publications each. These were autologous fibroblasts (Ishihara et al., 2009, 2010), allogeneic BMSCs (Wegman et al., 2012; Loozen et al., 2015), allogeneic adipose-derived mesenchymal stem cells (ADSCs) (Lin et al., 2015; Kim et al., 2018), and human fetal osteoblasts (Wehrhan et al., 2012, 2013). Autologous ADSCs were used by one publication (Chen et al., 2010). Thirteen publications did not use cells as a part of their gene therapy (Bonadio et al., 1999; Backstrom et al., 2004; Egermann et al., 2006a, b; Park et al., 2007; Zhang et al., 2007, 2009; Ishihara et al., 2008; Lutz et al., 2008; Santoni et al., 2008; Kroczek et al., 2010; Southwood et al., 2012; Bez et al., 2017). See Table 3 for details of the cells used by the publications. Doses were highly variable and reported in several different and non-comparable ways depending on several factors including vector, use of cells, use of scaffolds/constructs, and *in vivo* or *ex vivo* application. Frequently, scaffold-based applications (largely *ex vivo*) did not provide information regarding multiplicity of infection and/or gene transfer efficiency. Sixteen publications failed to provide one or both of these pieces of information (Chang et al., 2003a, b, 2010; Dai et al., 2005; Zhang et al., 2007, 2009; Lian et al., 2009; Chen et al., 2010; Xiao et al., 2010; Castro-Govea et al., 2012; Wegman et al., 2012; Wehrhan et al., 2012, 2013; Lin et al., 2015; Loozen et al., 2015; Kim et al., 2018). The picture is further confused by several publications that formally used *in vivo* approaches but added unmodified *ex vivo* cells to their gene-activated scaffolds shortly before implantation. In some cases, the number of cells was provided, but the quantity of vector in the scaffold was not (Zhang et al., 2007; Zhang et al., 2009). In a handful of cases, the number of cells applied to each scaffold was either not measured or not clearly provided (Castro-Govea et al., 2012; Loozen et al., 2015; Liu et al., 2016; Kim et al., 2018). Non-viral *in vivo* approaches accounted for five publications. The two publications that used naked DNA used quantities measured in tens of milligrams (Bonadio et al., 1999; Backstrom et al., 2004), while the three publications that used carrier agents used either 12 μg (Park et al., 2007; Lutz et al., 2008) or 1 mg (Bez et al., 2017). See Table 4 for dosage information. Unfortunately, it was not possible to make accurate estimates of the dose in terms comparable with human clinical doses (mg DNA/kg bodyweight) due to lack of information regarding the animals used. Often, only the range of weights of animals was provided with no measurement of central tendency, and furthermore, authors frequently did not provide information regarding animal breed or variety to allow a more accurate estimation. Estimated doses varied hugely, from 5.33 × 10^–5^ to 7.69 mg/kg (data available on request). All four scaffold-free viral *in vivo* approaches used roughly similar amounts of vector, between 1 × 10^11^ and 5 × 10^11^ particles (Egermann et al., 2006a, b; Ishihara et al., 2008; Southwood et al., 2012). Dosage estimates in particles/kg body weight were calculated based on estimated animal weight. Again, measures of central tendency were not provided by three of these publications, so rough estimates were made from the range of animal weights. Estimated viral doses were much more consistent than those of non-viral approaches, ranging between ∼4.7 × 10^8^ and ∼1.6 × 10^9^ particles/kg (data available on request).

### Outcome Parameters and Analyses

**FIGURE 10:**
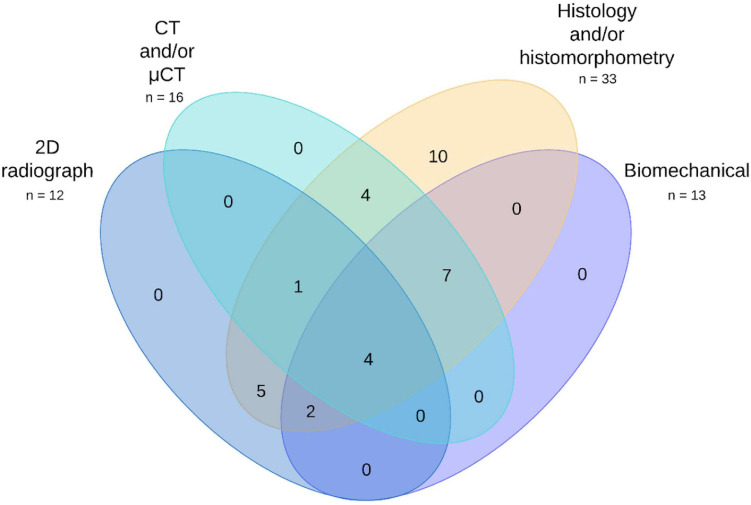
Venn diagram of the most commonly used preclinical evaluation techniques across the publication set. See Supplementary Data for full details.

Four families of techniques were commonly used to investigate regenerated tissue. These were 2D radiograph, CT and μCT, histology and biomechanical testing (see Figure 10). Two-dimensional radiographs were used by 12 publications (Bonadio et al., 1999; Backstrom et al., 2004; Dai et al., 2005; Xu et al., 2005; Egermann et al., 2006b; Ishihara et al., 2008, 2009; Santoni et al., 2008; Lian et al., 2009; Chen et al., 2010; Castro-Govea et al., 2012; Southwood et al., 2012) and not used by 21 (Chang et al., 2003a, b, 2009, 2010; Egermann et al., 2006a; Park et al., 2007; Zhang et al., 2007, 2009; Lutz et al., 2008; Ishihara et al., 2010; Kroczek et al., 2010; Xiao et al., 2010; Wegman et al., 2012; Wehrhan et al., 2012, 2013; Deng et al., 2014; Lin et al., 2015; Loozen et al., 2015; Liu et al., 2016; Bez et al., 2017; Kim et al., 2018). CT or μCT was used by 16 publications (Chang et al., 2003a, b, 2009, 2010; Backstrom et al., 2004; Dai et al., 2005; Egermann et al., 2006a, b; Ishihara et al., 2008, 2009, 2010; Xiao et al., 2010; Deng et al., 2014; Lin et al., 2015; Bez et al., 2017; Kim et al., 2018) and not used by 17 (Bonadio et al., 1999; Xu et al., 2005; Park et al., 2007; Zhang et al., 2007, 2009; Lutz et al., 2008; Santoni et al., 2008; Lian et al., 2009; Chen et al., 2010; Kroczek et al., 2010; Castro-Govea et al., 2012; Southwood et al., 2012; Wegman et al., 2012; Wehrhan et al., 2012, 2013; Loozen et al., 2015; Liu et al., 2016). Histology was used by all 33 publications. Various forms of biomechanical testing were used by 13 publications (Chang et al., 2003a, b, 2009, 2010; Dai et al., 2005; Xu et al., 2005; Egermann et al., 2006a, b; Ishihara et al., 2008, 2009; Lian et al., 2009; Lin et al., 2015; Bez et al., 2017), and not used by 20 (Bonadio et al., 1999; Backstrom et al., 2004; Park et al., 2007; Zhang et al., 2007, 2009; Lutz et al., 2008; Santoni et al., 2008; Chen et al., 2010; Ishihara et al., 2010; Kroczek et al., 2010; Xiao et al., 2010; Castro-Govea et al., 2012; Southwood et al., 2012; Wegman et al., 2012; Wehrhan et al., 2012, 2013; Deng et al., 2014; Loozen et al., 2015; Liu et al., 2016; Kim et al., 2018). A handful of other analysis techniques were used. These were microradiography by four publications (Park et al., 2007; Lutz et al., 2008; Kroczek et al., 2010; Southwood et al., 2012); calcein staining by three publications (Zhang et al., 2007, 2009; Xiao et al., 2010); immunohistochemistry for osteogenic markers by three publications (Bonadio et al., 1999; Wehrhan et al., 2013; Loozen et al., 2015); and PET/CT (Lin et al., 2015), radionuclide imaging (Chen et al., 2010), and single-photon imaging (Chen et al., 2010) by one publication each. The remaining 24 publications did not employ any additional methods. Outcome parameter information is provided in full in Supplementary Table 2.

The investigation of three additional factors was taken as a yardstick for closeness to clinical translation. These factors were immunological response, therapy persistence, and vector biodistribution. Note that many publications investigated vector persistence *in vitro*; however, only attempts to investigate persistence and the other factors *in vivo* were included at this stage. Eleven publications made some attempt to investigate the immune response to their therapy (Backstrom et al., 2004; Xu et al., 2005; Egermann et al., 2006a, b; Ishihara et al., 2008; Chen et al., 2010; Castro-Govea et al., 2012; Southwood et al., 2012; Wehrhan et al., 2012, 2013); however, this was often only an assessment of histology. Twenty-two publications made no reference to the immune response to their therapy whatsoever. Nine publications attempted to investigate transgene or vector persistence (Bonadio et al., 1999; Chang et al., 2003a, b; Egermann et al., 2006a, b; Santoni et al., 2008; Ishihara et al., 2010; Wegman et al., 2012; Bez et al., 2017), while 23 did not. Only five publications investigated vector biodistribution (Bonadio et al., 1999; Egermann et al., 2006a, b; Ishihara et al., 2008; Bez et al., 2017), with the remaining 28 failing to do so. Information regarding factors vital for translation is provided in Supplementary Table 1.

All 33 manuscripts used histological techniques that are expensive and difficult to perform. See Table 5 for a summary of methods used. Histological analyses were carried out according to different protocols; therefore, the obtained results have to be considered to be of different reliability. Six of the 33 publications (18%) used only H&E staining (with an additional two using Giemsa–eosin staining), and another two studies used only toluidine blue staining. Multicolor techniques allowing more accurate differentiation of elements in the newly formed tissues—Van Gieson bichrome, Masson trichrome, Masson–Goldner trichrome, and Gomori and Sanderson trichrome—were used in 13 publications (39.4%).

**TABLE 5:** List of histological procedures performed in analyzed studies.

Parameters	*n*	*%*
Histological examination	33	100
Morphometric examination	20	60.6
Undecalcified sections in methyl methacrylate	19	57.6
Static morphometric indicators	20	60.6
Dynamic morphometric indicators	8	24.2
Von Kossa staining	4	12
Immunohistochemical study	11	33.3

Morphometry and statistical analysis were performed only in 60.6% (*n* = 20) of studies (Backstrom et al., 2004; Dai et al., 2005; Xu et al., 2005; Egermann et al., 2006a, b; Park et al., 2007; Zhang et al., 2007, 2009; Ishihara et al., 2008, 2009, 2010; Lutz et al., 2008; Santoni et al., 2008; Xiao et al., 2010; Wegman et al., 2012; Wehrhan et al., 2012, 2013; Deng et al., 2014; Loozen et al., 2015; Liu et al., 2016), and in five cases, it did not assess the bone tissue but additional parameters such as the number of blood vessels, the severity of inflammation, and the linear sizes of bone callus. Nineteen studies (57.6%) used a complex technique for sectioning non-decalcified bone that had been previously placed in methyl methacrylate or its analogs, Technovit 9100 and Osteo-Bed (Polysciences) (Backstrom et al., 2004; Egermann et al., 2006a, b; Park et al., 2007; Zhang et al., 2007, 2009; Ishihara et al., 2008, 2009, 2010; Lutz et al., 2008; Santoni et al., 2008; Xiao et al., 2010; Wegman et al., 2012; Wehrhan et al., 2012, 2013; Deng et al., 2014; Loozen et al., 2015; Liu et al., 2016; Bez et al., 2017), but only eight of them (24.2% of all publications) then used the methods for assessment of dynamic indicators of bone formation [measure mineralizing surface (MS), mineral apposition rate (MAR), and bone formation rate (BFR)] (Backstrom et al., 2004; Zhang et al., 2007, 2009; Santoni et al., 2008; Ishihara et al., 2009, 2010; Xiao et al., 2010; Loozen et al., 2015), the main reason for using this technique. Static morphometric indicators were evaluated in all experiments where morphometry was performed, and these included the following: the proportion of bone tissue (BT/TV); the percentage of cartilage and connective tissue in the newly formed tissues; the areas of remaining bone substitute; and the length of bone tissue in direct contact with bone substitute (the percentage of available scaffold perimeter in contact to bone) or to a metal implant (bone-to-implant contact).

Von Kossa staining, a method commonly used to characterize mineralization of tissues, was used in 12% of the publications (*n* = 4) (Chang et al., 2003a, b, 2010; Castro-Govea et al., 2012). It is important to note that this method cannot be used to quantify mineralization and may mask important features of tissue reactions due to black color on histological slides. For these reasons, it is of limited value when assessing regeneration.

Immunohistochemical technique for detecting viral particles, expression of the protein encoded by transgene, and markers of cell differentiation within the newly formed tissues were used in 33.3% of all studies (*n* = 11) (Bonadio et al., 1999; Chang et al., 2003a, b; Park et al., 2007; Santoni et al., 2008; Kroczek et al., 2010; Wegman et al., 2012; Wehrhan et al., 2012, 2013; Lin et al., 2015; Loozen et al., 2015).

### SYRCLE Risk of Bias Tool for Animal Study Assessment

The SYRCLE RoB assessment revealed that a large majority of the publications had not considered or mentioned most factors covered by the tool (detailed analysis available on request). Sequence generation (that is, the assignment of animals to groups) was only randomized by six publications, with none of these stating which randomization method was used. Six additional publications indicated they had randomized treatment sites within animals or used a blocking strategy, although again none stated which randomization method was used. The remaining 21 publications made no indication that randomization had been considered. Only one publication clearly indicated that animal baseline characteristics had been considered during group allocation. Two others stated that they had tried to balance groups based on only a subset of our defined baseline characteristics or additional factors. The remaining 30 publications did not address group balance. The assessment factors of group allocation concealment, random animal housing, investigator blinding at the treatment stage, and random assessment at the outcome stage were not considered by any publications. Investigator blinding at the analysis stage was performed by two publications for portions of their data. No publications blinded investigators for all of their data. The remaining 31 papers did not indicate that blinding at the data analysis stage had been considered. Thirteen publications clearly addressed the subject of incomplete experimental outcomes, either stating none had occurred or listing their number and nature. Only one publication clearly stated that there had been incomplete outcomes during the study but did not provide the number or nature of these events. Nineteen publications did not mention incomplete outcomes. While it is possible none occurred in these studies, it cannot be safely assumed. Selective outcome reporting (not providing data that the methods section indicates was collected, or clear gaps in the methods section) was common, with 29 publications identified to have failed to provide some information. The large majority of these cases were minor, with common omissions including failing to provide non-significant *p*-values or histology or radiographic images for every time point and group. More serious failings included not clearly indicating numbers of animals used or group sizes, providing no radiographic images despite extensive use of the technique and providing no histology images despite extensive use of the technique. Only four publications were found to have provided all expected data and information. Several other sources of bias not directly covered by the assessment were identified across the publication set. Fourteen publications did not indicate that they had tested if their data were normally distributed prior to applying a parametric statistical test. Rarer issues included exceptionally small group sizes (*n* = 1 or 2) and use of animals for simultaneous experiments not included in the publication. The full results of the RoB assessment are available on request.

## Discussion

The publications reviewed here cover a wide range of approaches to gene therapy for bone regeneration in large animal models. This is both a blessing and a curse. It is clearly beneficial to attempt different approaches to establish which methods may be the most effective. However, there is currently such little consistency between studies that it is very difficult to compare effectiveness. Some simple trends across the literature can be clearly seen, for example, aspects of vector design, the use of orthotopic test sites, and bone analysis methods. However, almost all other elements of experimental design are highly variable, including crucially model species, defect sites, and specifics of analysis techniques.

While it is difficult to make generalizations, it seems that species choice was primarily influenced by practicality and researcher familiarity rather than advantages of the model system. Defects in horses were typically small and never larger than 15 mm (Southwood et al., 2012), while defects in goats were never smaller than 21 mm (Xu et al., 2005) despite being a substantially smaller species. A similar situation seemed to have occurred in choice of defect site, with little rationale provided as to why various locations where chosen. If we consider a model system to be a species, bone, and defect type, no particular model was used by more than a handful of publications. Porcine cranial or calvarial defects were the most popular, with seven publications (Chang et al., 2003b, 2009, 2010; Park et al., 2007; Wehrhan et al., 2012, 2013); however, even this group was split between pigs and mini-pigs, and defect locations were not described in a consistent manner (clear indications of where defects were created were not provided, with the terms skull, cranium, calvarium, frontal bone, and frontal skull all used by various publications). Frequently, publications used a combination of species, bone, and defect type that was unique. Similarities further break down when experimental techniques are considered. Even within a defined model system, publications used unique combinations of defect size, fixation method, vector, gene/s, cells, scaffold, dose, experimental length, data collection methods, and time points. The importance of defect fixation should not be overlooked, as fixation method and quality influence fracture healing (O’Sullivan et al., 1989; Claes et al., 1999; Hak et al., 2010). This will likely influence experimental results, and the performance of novel approaches likely should be tested using various different fixation approaches prior to translation. Additionally, the precise biomechanics of each defect location are unique and highly dependent on surgical technique. Even in areas that seem to be consistent, there were differences that lead to difficulty in comparing results. For example, approximately one third of publications used 2D radiographs to investigate rates of defect closure, but time points and measurement methods varied considerably between publications. A similar situation was seen with histology and histomorphometry, used by all the publications in the set, where different authors often used completely different and often highly qualitative evaluation parameters. Furthermore, there were additional experimental procedures that were not reviewed (though most often not provided by the publications) that are likely to have some influence on results. The most important was thought to be the specifics of the surgical methods used (e.g., treatment of the periosteum), details of vector design other than transgene and promoter, and the length of time between modification and implantation of cells for *ex vivo* approaches. Consequently, a direct comparison of results was not possible between the large majority of publications in the set, and even among those that were relatively similar, it was thought to be unproductive due to the numerous differences in experimental design. This made it impossible to thoroughly answer the sub-questions posed at the start of the review.

Despite difficulties in the comparison, there is a general trend of positive results among the set. Near universally, some form of improvement versus control groups was seen, with only two publications showing unambiguously negative results (Santoni et al., 2008; Southwood et al., 2012). It is of course possible that this could be due to positive publication bias, which has been shown to be problematic across a wide swathe of fields and at both investigative and editorial levels (Olson et al., 2002; Hopewell et al., 2009; Mlinarić et al., 2017). Despite this, it seems possible to say that at a very broad level gene therapy does seem able to improve bone regeneration at a proof-of-concept level in these model species. This does provide an answer to the primary question of the review but is admittedly rather unsatisfying.

The generally positive results found by the first portion of the review are called into question by the RoB assessment, which found that investigators are universally doing a poor job of avoiding bias. Of the nine core factors covered by the assessment, no publication satisfactorily addressed more than two, and there was no indication that many of the factors were considered by any of the publications at all. The only two factors considered by a substantial fraction of the set were incomplete outcome reporting and group randomization; and even so, no publications reported randomization methods satisfactorily. In terms of additional factors, the most serious was thought to be potential statistical errors. Though many publications used parametric tests, many failed to check if such approaches were appropriate for their data. Overall, it would seem that researchers are either unaware of or unconcerned by the risks of bias, which can potentially lead to erroneous positive results and cumulatively do great harm to the progress of the field if widespread. For example, in the field of stroke, it has been reported that apparent substantial improvements in outcome can be largely attributed to bias, leading to a great deal of wasted time and resources pursuing treatments that were actually much less effective than first thought (Macleod et al., 2009). Bias is also likely an important factor in the present reproducibility crisis seen throughout biological science and biomedicine (Nuzzo, 2015). Based on the results of this review, it seems that the field of gene therapy for bone regeneration is likely suffering from the same problems as many others. A widespread and concerted push to improve study design in regard to bias is clearly required.

### Technology Readiness Assessment

In terms of technology readiness and closeness to translation, it seems that there is still some way to go. While results were generally positive, factors vital for gene therapy translation were routinely ignored. For the purposes of this review, the three factors of immune response, persistence of transgene expression, and biodistribution were taken as a yardstick for closeness to translation. Very few publications attempted to investigate these factors in any capacity, and those that did typically did not perform a thorough investigation (see Supplementary Table 1). Consequently, the reviewed approaches fit within TRL 4 (see Table 1 and Supplementary Table 4). TRL 5 would require in-depth safety studies, which have not been performed in these models. At the very least, more effort needs to be taken to fully characterize where and for how long therapies are working and possible immune responses. Alongside these, several additional factors should be more thoroughly investigated. While many publications demonstrated improved rates of union when compared with controls, the quality of the bone was highly variable or not adequately investigated. Biomechanical and μCT results were most useful in this regard, as they produce quantitative and potentially more comparable data. Several publications showed that their regenerated bone had comparable mechanical properties to native tissue (Chang et al., 2003a, b; Ishihara et al., 2008; Lian et al., 2009; Bez et al., 2017). Other publications found that despite improvements compared with no treatment controls or scaffold-only treatments, the regenerated bone showed significantly worse properties than native tissue (Dai et al., 2005; Xu et al., 2005; Lin et al., 2015). One publication used these methods but failed to compare with native tissue (Ishihara et al., 2009), while most failed to use them at all. A further criticism is the lack of comparison with existing and alternative treatments. Few studies investigated how gene therapy compared with autologous bone graft or recombinant growth factors (Park et al., 2007; Lutz et al., 2008; Kroczek et al., 2010). Only one publication, Bez et al. (2017) demonstrated that the tissue regenerated in their experiment had properties comparable with those of autograft controls across a variety of techniques including crucially mechanical testing. This is likely the clearest demonstration of non-inferiority yet provided in the field; however, it is currently a lone outlier. Others should follow the example of this publication when attempting to demonstrate the non-inferiority of their own approaches.

### Recommendations for Future Preclinical Work

To improve the comparability of research in the field and help bring gene therapy for bone regeneration closer to translation, we suggest that several principles of experimental design, large animal models, and investigative approaches should be defined and broadly adhered to by the research community. This would help to reduce bias and allow comparisons to be more easily drawn between different studies without restricting investigators to particular therapeutic approaches. In the following section, we propose potential standards in these areas. Our recommendations here are summarized in Supplementary Figure 1. Furthermore, as discussed by Iglesias-López et al. (2019) and in *Outlook and Current Status Regarding Clinical Studies and Authorized Clinical Gene Therapeutics for Orthopedics* section, it is essential to clearly classify the type of drug developed and applicable translational pathway as well as to perform early engagement with regulatory authorities to inform translation-enabling preclinical studies, ensuring that study design and data procurement would either support or directly feed into preclinical data packages required for investigational new drug (IND)-enabling studies prior to clinical trials.

#### Study Design and Data Reporting

We encourage investigators to adhere to the “Animal Research: Reporting of *In Vivo* Experiments” (ARRIVE) guidelines laid out by the United Kingdom National Centre for the replacement, refinement, and reduction of animals in research (NC3Rs) (Kilkenny et al., 2010). Additional guidance should be taken from the “Planning Research and Experimental Procedures on Animals: Recommendations for Excellence” (PREPARE) guidelines from Norecopa, Norway’s National Consensus Platform for the advancement of the 3Rs. We would also recommend that researchers consult the SYRCLE RoB tool to further inform themselves of reporting expectations (Hooijmans et al., 2014). While ARRIVE and the SYRCLE RoB tools are not formally experimental design guides, they can both act as useful sources of information for method and data reporting expectations and therefore inform design considerations required to meet them. We would also recommend investigators examine the relevant standards from the International Organization for Standardization (ISO) to inform themselves as to the knowledge required before beginning the process of translating an approach. While there are presently no ISO standards specifically focusing on gene therapies, many from the ICS 11.100.20: “Biological evaluation of medical devices” family are relevant to GAMs. ISO 10993-1:2018 “Evaluation and testing within a risk management process,” ISO 10993-2:2006 “Animal welfare requirements,” ISO 10993-6:2016 “Tests for local effects after implantation,” and ISO 10993-11:2017 “Tests for systemic toxicity” all contain relevant information. Investigators pursuing cell-based therapies should also consult ISO 13022:2012 “Medical products containing viable human cells—Application of risk management and requirements for processing practices.”

To investigate non-inferiority in comparison with current treatment approaches, we recommend that investigators always include a group treated with the current clinical gold standard, autologous bone graft. Investigators could also include a group treated with a recombinant version of their transgene as an additional positive control.

#### Suggested Animal Model Systems

We propose two different model systems, for load-bearing and non-load-bearing defects.

Load bearing: Sheep, femoral mid-diaphysis full-thickness osteotomy/segmental defect.

Non-load bearing: Mini-pig, calvarial full-thickness oval defect.

For load-bearing defects, we have selected the sheep femoral defect. While it could be argued that pigs are more representative of human bone in some regard (Reichert et al., 2009; Li et al., 2015; Wancket, 2015), sheep are of a comparable weight and have bones of a similar size to humans and are generally considered easier to work with. Additionally, they are a well-established model for long bone load-bearing defects in the regeneration of critical bone defects using other approaches (McGovern et al., 2018). Indeed, other investigators have already suggested sheep for standardized testing of biomaterials in large animals, although using a different defect location and type (Ferguson et al., 2018). For non-load-bearing defects, we have selected mini-pigs for a standard model species. The similarities between pig and human bone have been previously mentioned, and mini-pigs are easier to handle than their larger cousins. Additionally, this review has revealed that porcine calvarial defects are currently the most popular model system in the field; therefore, continued use of the model presents practical advantages.

As we have previously stated in this review, there is no clearly superior animal model for bone regeneration research, and as such, our recommendations here are fairly arbitrary. We are aware of the irony of suggesting our own arbitrary guidelines after criticizing authors for not providing adequate justification for model choice; however, we hope that the very act of standardization could prove highly beneficial to the field. If these suggestions prove to be impractical, we hope they will at least spur discussion of more appropriate standardized options.

#### Analysis Techniques

We recommend authors place a greater emphasis on quantitative techniques such as biomechanical testing and μCT imaging and reduce their reliance on qualitative and semi-quantitative approaches such as histology and 2D radiographs. Quantitative techniques are vital for formally demonstrating the non-inferiority of novel approaches to the existing clinical gold standard and would allow a more valuable comparison of data between studies. Qualitative and semi-quantitative methods still have value as supporting information, and standardization of approaches would further improve their usefulness. Histology can provide extremely useful information that is not provided by other methods if good standards of practice, blinding, and evaluation are maintained. Novel, automated methods in digital histology and machine learning approaches certainly have the potential to revolutionize this approach and provide reliable results in the future (Aeffner et al., 2019). Undoubtedly, these techniques provide useful information, and we do not want to discourage investigators from their use; however, we advise against over-reliance. A combination of various quantitative and qualitative techniques will provide the best picture of examined tissue, adding real value to the study.

In summary, we would like to suggest possible standards for improving the quality, comparability, and reproducibility of outcome measures and procedures for future preclinical work in the field of orthopedic gene therapy (Supplementary mindmap in Supplementary Figure 1) in addition to the recommended use of model systems previously suggested.

### Outlook and Current Status Regarding Clinical Studies and Authorized Clinical Gene Therapeutics for Orthopedics

To date, gene therapy for orthopedics has seen little use in the clinic, with only a single case of clinical translation. While numerous clinical trials have been carried out for osteoarthritis (Evans et al., 2018) and different types of myodystrophies, only two clinical studies have been initiated for bone regeneration (trial numbers NCT02293031 and NCT03076138), both in the Russian Federation. The first of these, for a gene-activated bone substitute based on collagen–hydroxyapatite scaffold containing a VEGFA plasmid, was started in November 2014. Despite the positive results, the study was withdrawn for commercial reasons (Bozo et al., 2016). The same group proceeded with another clinical trial aiming to investigate another gene-activated bone substitute based on an octacalcium phosphate scaffold and a *VEGFA* plasmid. The results of this study were reported at the TERMIS-EU Meeting in Rhodes 2019 (Bozo et al., 2019). Twenty patients with alveolar ridge atrophy and or mandible defects were successfully treated within 6 months, and no adverse events were observed. Approval for clinical use of this approach in the Russian Federation was granted in April 2019 and is now available for oral and maxillofacial surgeons. Between these two clinical trials, another group based in Kazan, Russia, published a clinical case report of a successful treatment of a patient with ulnar pseudarthrosis using the GAM based on demineralized bone allograft and a dual cassette plasmid encoding *VEGFA* and *BMP2* (Masgutov et al., 2017).

Gene therapy for bone regeneration is now clearly on its way to clinical practice; however, the clinical data are currently limited, and no human trials have been performed outside the Russian Federation. Continued successful clinical translation of regenerative orthopedic gene therapy products is limited by several factors closely associated with the particular complexities of different regulatory environments. An excellent review of EMA and FDA frameworks for advanced therapy medicinal products (ATMPs) has been provided by Iglesias-López et al. (2019). This diversity in definitions, legal recommendations, and requirements for the translation of gene therapies for regenerative medicine applications across different regulatory framework increases risk and cost and leads to extensive translational timeframes for gene therapy developers in this space. In both jurisdictions, all types of advanced therapies discussed in this review would fall under the classification of medicines/biological products with differences in terms of further granularity and classification [e.g., gene therapy medicinal product (GTMP), combined ATMP (cATMP), and CGT products]. The extent and effect on translation of global regulatory differences and the diversity in interpretation and preclinical and clinical study requirements of orthopedic gene therapy approaches can be illustrated using the specific example of GAM translation using a biomaterial in conjunction with a gene therapy vector. In both EMA and FDA jurisdiction, this approach would be considered an ATMP. GAMs, for example, are considered to be combination products under FDA regulations. Similarly, under EMA regulations, they fulfill the definition of a GTMP as defined in Article 2(1) of Regulation (EC) No. 1394/2007 and a cATMP as defined in Article 2(1) of Regulation (EC) No. 1394/2007 due to the incorporation of a scaffold. The strict regulatory environments in the United States and EU have led to relatively slow clinical translation pathways, although novel expedited pathways/classifications are available for accelerated translation (e.g., RMAT in the United States). Furthermore, differences in terms of definitions, classification, and regulatory requirements between EMA and FDA regulatory legislation mostly require separate, independent clinical trials and preclinical data (e.g., IND submission requirements in the United States) procurement for each jurisdiction. This is further complicated by the oversight of clinical trials by national regulatory authorities [e.g., Medicines and Healthcare products Regulatory Agency (MHRA) in the United Kingdom] in individual member states in the EU. In EMA regulatory space, a centralized procedure for market authorization is mandatory for ATMPs. Therefore, parallel deployment of a novel gene therapy in the global market within the United States and EU requires significant resources and time and cannot be expedited, as for now no harmonized procedure is available covering both regulatory spaces. The regulatory requirements for translation of somatic gene therapeutics are, however, substantially different inside the Russian Federation. The advantage for rapid, accelerated translation of a novel gene therapeutic, in particular for regenerative applications if developed as a combination device with a biomaterial, is that if a material contains biologically active components, the combination product is still defined as a medical device. Only the biological component, in this case the plasmid DNA for GAMs, needs to be separately registered as a drug. The first treatment to be approved in this way was a gene therapy drug comprising plasmid DNA encoding *VEGFA* (“Neovasculgen^®^,” HSCI, Russia) for the treatment of chronic lower limb ischemia. After three clinical trials (trial numbers NCT02369809, NCT02538705, and NCT03068585), the drug was approved in the Russian Federation in 2011 (Deev et al., 2017) as a gene therapy. This approval subsequently allowed the use of the authorized Neovasculgen^®^ product in the Nucleostim GAM combination device product, which is now undergoing clinical trials in the Russian Federation or has already completed trials depending on the indication (NCT03076138 and NCT02293031). This peculiarity of the Russian regulatory environment for combination products allowed a phased approach wherein an authorized gene therapy drug can be subsequently combined with a biomaterial and repurposed as a GAM. Thereby, for the new indication of orthopedic gene therapy, an accelerated path to translation can be pursued wherein the medical device pathway can be followed even if the resulting product is a cATMP. This means, however, that for new approaches that are developed, all components would need to follow separate translational pathways (i.e., the gene therapeutic biologic component used follows drug translation pathway and the materials have to follow the device route). Therefore, this can only be regarded as an advantage for accelerated translation if there is already a separate gene therapeutic that can be repurposed and integrated to a biomaterial for the subsequent indication.

This example illustrates that there are still significant worldwide differences in regulatory approaches to ATMP translation and that orthopedic gene therapy in particular encompasses a complex array of different approaches that can be classed as either GTMPs/CGTs or combination approaches with different terminologies, definitions, and regulatory requirements. Even relatively closely aligned regulatory frameworks such as EMA and FDA exhibit significant differences that result in the requirement of independent translational regulatory pathways to market authorization if one desires to bring an orthopedic gene therapy to the clinic for patient benefit. Ultimately, it would be highly desirable to facilitate harmonization and convergence (Iglesias-López et al., 2019) of different regulatory environments dealing with ATMPs to enable accelerated translation of these promising approaches in the future and facilitate cross-jurisdiction accreditation of preclinical and clinical study data. This would not only facilitate the successful deployment of these future therapies within the orthopedic space but also eliminate or at least ameliorate the major bottleneck of complex regulatory requirements and highly diverse global environments. Due to the nature of the complex matter of ATMPs and the current trend of global political compartmentalization and fragmentation, it is difficult to predict whether this would be a far-fetched possibility or an illusion; it would, however, be a clear driver to make clinical gene therapy a more widespread routine intervention in the future.

## Data Availability Statement

The original contributions presented in the study are included in the article/Supplementary Material, further inquiries can be directed to the corresponding author/s.

## Author Contributions

PW wrote the major part of the manuscript and performed the systematic review. IB wrote the clinical trials part and edited and reviewed the manuscript. TB was the second reviewer performing the systematic review. PJ wrote the program and performed the machine reading analysis. EJ contributed to the manuscript text and edited and reviewed. RD performed the systematic analysis of histological methods, contributed to the manuscript text, and reviewed and edited the manuscript. PG participated in conceptualization, contributed to the clinical aspects of the manuscript and reviewed and edited the manuscript. GF proposed the manuscript, conceptualized its contents, supervised PW and TB, wrote the translational part of the manuscript, and reviewed and edited and handled submission. All authors contributed to the article and approved the submitted version.

## Conflict of Interest

IB is the CEO of the company Histograft, LLC. PJ is the CEO of the company Into Numbers Data Science GmbH. GF works as *pro bono*, non-salaried consultant for Histograft, LLC. The remaining authors declare that the research was conducted in the absence of any commercial or financial relationships that could be construed as a potential conflict of interest.

## Footnotes

1 https://www.nltk.org/index.html
