# Supplementary material for: Systematic Review of the Preclinical Technology Readiness of Orthopedic Gene Therapy and Outlook for Clinical Translation
Source: Front Bioeng Biotechnol. 2021 Mar 17;9:626315. doi: 10.3389/fbioe.2021.626315 (PMC8011540; doi:10.3389/fbioe.2021.626315)
Supplement: Supplementary file 1 [file Data_Sheet_1.DOCX]

## Supplementary information

Figure S1: Mind map of recommended improvements for study design and reporting for preclinical studies across the field.


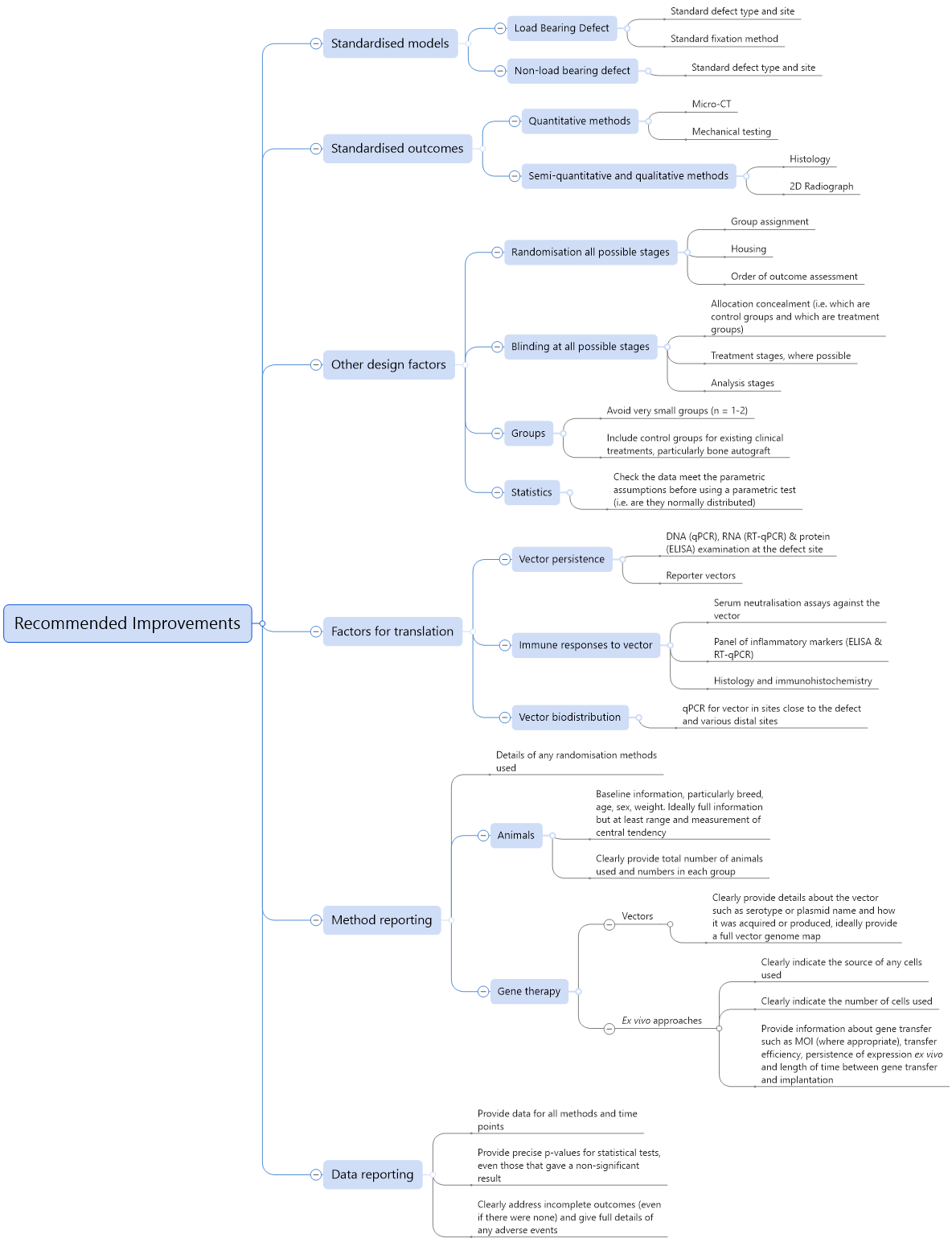


| Authors  **Additional methods tables** | Immunological response | Persistence of expression | Biodistribution |
| --- | --- | --- | --- |
| (Backstrom et al., 2004) | Little evidence of inflammation in histology | NA | NA |
| (Bez et al., 2017) | NA | RNA and protein gone by d10 at defect | qPCR for BMP-6 in various organs. No significant off target expression seen |
| (Bonadio et al., 1999) | NA | DNA and RNA detectable at week 6, but no protein | Blood tests to check for circulating transgene, never "above normal". No evidence of ectopic bone formation in any other tissue, not stated how this was examined. |
| (Castro-Govea et al., 2012) | Immunology not directly investigated but vector liver toxicity tested by investigating prothrombin time and concentration of various serum markers | NA | NA |
| (Chang et al., 2003b) | NA | Immunohistochemistry for AdV shows no evidence of residual vector at defect site after 3 months, but no investigation of gene expression | NA |
| (Chang et al., 2010) | NA | NA | NA |
| (Chang et al., 2003a) | NA | Immunohistochemistry for AdV shows no evidence of residual vector at defect site after 6 weeks, but no investigation of gene expression | NA |
| (Chang et al., 2009) | NA | NA | NA |
| (Chen et al., 2010) | Little inflammation observed in histology | NA | NA |
| (Dai et al., 2005) | NA | NA | NA |
| (Deng et al., 2014) | NA | NA | NA |
| (Egermann et al., 2006a) | Serum neutralisation assays at days 14,28,56. Neutralising antibodies detected at d14 but not at later points | Separate pilot study with luciferase reporter virus indicates rapid drop off after wk1, greatly reduced expression is still detectable at wk6 | Luciferase pilot study indicates that soft tissues near defect were also transduced, no information for distal sites |
| (Egermann et al., 2006b) | Serum neutralisation assay for anti-Ad5 antibodies. Antibodies were present and at highest conc. at week 2. Also performed indirect ELISA for anti-BMP2 antibodies in serum. Significantly more inflammatory cells in BMP2 treated sites. OD measurements significantly higher in treated animals, suggesting presence of circulating anti-BMP2 antibodies. | Tested in luciferase control. Detection by light production lost between 5 and 6 weeks in defect. | Tested in luciferase control. Low levels seen at distant sites in 2 animals |
| (Ishihara et al., 2008) | Peripheral venous blood hemogram, Serum neutralisation assay: increased vs controls but not correlated with outcome (statistical method not stated) | NA | qPCR indicates no off target CMV |
| (Ishihara et al., 2009) | NA | NA | NA |
| (Ishihara et al., 2010) | NA | Only tracked at d2 in the ilium defects. BMP seen but no GFP | NA |
| (Kim et al., 2018) | NA | NA | NA |
| (Kroczek et al., 2010) | NA | NA | NA |
| (Lin et al., 2015) | NA | NA | NA |
| (Liu et al., 2016) | NA | NA | NA |
| (Loozen et al., 2015) | NA | NA | NA |
| (Lutz et al., 2008) | NA | NA | NA |
| (Park et al., 2007) | NA | NA | NA |
| (Santoni et al., 2008) | NA | No BMP or GFP protein in defect after 4 months by immunohistochemistry or western | NA |
| (Southwood et al., 2012) | Histology shows no inflammation | NA | NA |
| (Wegman et al., 2012) | Histology only. Indicates minimal immune response and no encapsulation at ectopic site. Larger immune response (foreign body) seen at orthotopic site, thought to be due to calcified alginate. | Immunohistochemistry indicates expression of His-tagged BMP-2 at point of sacrifice (16wks) | NA |
| (Wehrhan et al., 2013) | No macroscopic signs of inflammation | NA | NA |
| (Wehrhan et al., 2012) | Histology shows no inflammation | NA | NA |
| (Xiao et al., 2010) | NA | NA | NA |
| (Xu et al., 2005) | T-lymphocyte proliferation assay shows evidence of increased lymphocyte proliferation for 4 weeks. Plasma neutralisation assay shows increased humoral response in treatment groups for >17 weeks | NA | NA |
| (Zhang et al., 2009) | NA | NA | NA |
| (Zhang et al., 2007) | NA | NA | NA |
| (Lian et al., 2009) | NA | NA | NA |
| Table S1: Study metrics 4. Information regarding additional factors relevant to translation. | | | |

| Authors | Plain/Macroscopic 2D radiograph | CT and/or μCT | Histology and/or histomorphometry |  | Biomechanical | Other |
| --- | --- | --- | --- | --- | --- | --- |
| (Backstrom et al., 2004) | ✓ | ✓ | ✓ |  | 🗶 | NA |
| (Bez et al., 2017) | 🗶 | ✓ | ✓ |  | ✓ | NA |
| (Bonadio et al., 1999) | ✓ | 🗶 | ✓ |  | 🗶 | Immunohistochemistry (IHC) for osteogenesis markers |
| (Castro-Govea et al., 2012) | ✓ | 🗶 | ✓ |  | 🗶 | NA |
| (Chang et al., 2003b) | 🗶 | ✓ | ✓ |  | ✓ | NA |
| (Chang et al., 2010) | 🗶 | ✓ | ✓ |  | ✓ | NA |
| (Chang et al., 2003a) | 🗶 | ✓ | ✓ |  | ✓ | NA |
| (Chang et al., 2009) | 🗶 | ✓ | ✓ |  | ✓ | NA |
| (Chen et al., 2010) | ✓ | 🗶 | ✓ |  | 🗶 | Radionuclide bone imaging and single photon emission computerized tomography (SPECT) |
| (Dai et al., 2005) | ✓ | ✓ | ✓ |  | ✓ | NA |
| (Deng et al., 2014) | 🗶 | ✓ | ✓ |  | 🗶 | NA |
| (Egermann et al., 2006a) | 🗶 | ✓ | ✓ |  | ✓ | NA |
| (Egermann et al., 2006b) | ✓ | ✓ | ✓ |  | ✓ | NA |
| (Ishihara et al., 2008) | ✓ | ✓ | ✓ |  | ✓ | NA |
| (Ishihara et al., 2009) | ✓ | ✓ | ✓ |  | ✓ | NA |
| (Ishihara et al., 2010) | 🗶 | ✓ | ✓ |  | 🗶 | NA |
| (Kim et al., 2018) | 🗶 | ✓ | ✓ |  | 🗶 | NA |
| (Kroczek et al., 2010) | 🗶 | 🗶 | ✓ |  | 🗶 | Microradiography |
| (Lin et al., 2015) | 🗶 | ✓ | ✓ |  | ✓ | PET/CT |
| (Liu et al., 2016) | 🗶 | 🗶 | ✓ |  | 🗶 | NA |
| (Loozen et al., 2015) | 🗶 | 🗶 | ✓ |  | 🗶 | IHC for osteogenesis markers |
| (Lutz et al., 2008) | 🗶 | 🗶 | ✓ |  | 🗶 | Microradiography |
| (Park et al., 2007) | 🗶 | 🗶 | ✓ |  | 🗶 | Microradiography |
| (Santoni et al., 2008) | ✓ | 🗶 | ✓ |  | 🗶 | NA |
| (Southwood et al., 2012) | ✓ | 🗶 | ✓ |  | 🗶 | Microradiography, Dual x-ray absorptiometry |
| (Wegman et al., 2012) | 🗶 | 🗶 | ✓ |  | 🗶 | NA |
| (Wehrhan et al., 2013) | 🗶 | 🗶 | ✓ |  | 🗶 | IHC for osteogenesis markers |
| (Wehrhan et al., 2012) | 🗶 | 🗶 | ✓ |  | 🗶 | NA |
| (Xiao et al., 2010) | 🗶 | ✓ | ✓ |  | 🗶 | Calcein staining |
| (Xu et al., 2005) | ✓ | 🗶 | ✓ |  | ✓ | NA |
| (Zhang et al., 2009) | 🗶 | 🗶 | ✓ |  | 🗶 | Calcein staining |
| (Zhang et al., 2007) | 🗶 | 🗶 | ✓ |  | 🗶 | Calcein staining |
| (Lian et al., 2009) | ✓ | 🗶 | ✓ |  | ✓ | NA |
| Table S2: Study metrics 5. Information regarding bone investigation methods. | | | | | | |

**Literature search information**

Searches were conducted on three online databases: MEDLINE (via NCBI PubMed), BIOSIS citation index (via Clarivate Analytics Web of Science) and EMBASE/EMBASE Classic (via Wolters Kluwer OVID). Searches were performed in August 2019. Controlled vocabulary or curated subject terms for animal models, gene therapy and bone were used for all databases. For MEDLINE searches Medical Subject Headings (MeSH terms) were used without subheading restrictions, except in the case of the term “Animals” where subheadings were disabled. In the EMBASE search Emtree terms were used. For BIOSIS searches “concept codes” (CCs) and “major concepts” (MCs) headings were used. As BIOSIS CCs and MCs did not contain terms as specific to the topic of the review as those in other databases, terms were used to specify all topics in the “Bones, joints, fasciae, connective and adipose tissue” CC group and the “Skeletal System” MC. As this still led to rather broad search, a topic search for the search string “*(“gene therapy” OR “genetic therapy”) AND “animal model$” AND bone$*” was also performed. See Table S3 for a summary of the various search terms and reserved vocabulary used.

| Database  (Search engine) | Search string/s | Publications returned |
| --- | --- | --- |
| MEDLINE  (PubMed) | (“Models, Animal”[mesh] OR “Animals”[Mesh:NoExp]) AND (“genetic therapy”[mesh] OR “genetic vectors”[mesh] OR “gene transfer techniques”[mesh]) AND (“bone and bones”[mesh] OR “bone regeneration”[mesh] OR “fracture healing”[mesh]) | 1266 |
| BIOSIS citation index  (Web of Science) | Topic: (“gene therapy” OR “genetic therapy”) AND “animal model$” AND bone$  AND  Concept codes: 18001 OR 18004 OR 18006  AND  Major concepts: Molecular Genetics AND Skeletal System | 131 |
| EMBASE + EMBASE Classic  (OVID) | gene therapy/ and animal model/ and (bone/ or bone defect/ or bone injury/ or bone regeneration/) | 86 |
| Table S3: Listed search terms used in the literature search. In the EMBASE (OVID) search backslashes indicate Emtree terms, the curated subject headings for EMBASE. | | |

**Detailed technology readiness assessment frameworks**

| **TRL** | **Pharmaceutical (Biologics, Vaccines)** | **Medical Devices** |
| --- | --- | --- |
| 1 | Lowest level of technology readiness. Maintenance of scientific awareness and generation of scientific and bioengineering knowledge base. Scientific findings are reviewed and assessed as a foundation for characterizing new technologies. | Lowest level of technology readiness. Maintenance of scientific awareness and generation of scientific and bioengineering knowledge base. Scientific findings are reviewed and assessed as a foundation for characterizing new technologies. |
| 2 | Intense intellectual focus on the problem, with generation of scientific “paper studies” that review and generate research ideas, hypotheses, and experimental designs for addressing the related scientific issues. | Intense intellectual focus on the problem, with generation of scientific “paper studies” that review and generate research ideas, hypotheses, and experimental designs for addressing the related scientific issues. |
| 3 | Basic research, data collection, and analysis begin in order to test hypothesis, explore alternative concepts, and identify and evaluate critical technologies and components supporting candidate biologic/vaccine constructs research and eventual development of a candidate countermeasure. Research-scale process initiation and evaluation is conducted, as are studies to identify site(s) and mechanism(s) of action, potential correlates of protection for vaccines, and initial physical/chemical characterization of constructs. | Basic research, data collection, and analysis begin in order to test hypothesis, explore alternative concepts, and identify and evaluate component technologies. Initial tests of design concept and evaluation of candidate(s). Study endpoints defined. Animal models (if any) are proposed. |
| 4 | Non-GLP laboratory research to refine hypothesis and identify relevant data required for technological assessment. Exploratory study of critical technologies for effective integration into candidate biologic/vaccine constructs. Candidate biologic/vaccine constructs are evaluated in animal model(s) to identify and assess safety and toxicity, biological effects, adverse effects, and side effects. Assays, surrogate markers, and endpoints to be used during non-clinical and clinical studies to evaluate and characterize candidate biologic/vaccine constructs are identified. | Non-GLP laboratory research to refine hypothesis and identify relevant data required for technological assessment. Exploratory study of candidate device(s)/systems. Candidate devices/systems are evaluated in laboratory and/or animal models to identify and assess potential safety problems, adverse events, and side effects. Procedures and methods to be used during non-clinical and clinical studies in evaluating candidate devices/systems are identified. |
| 5 | Intense preclinical research. Identify manufacturing process amenable to cGMP-compliant production, identifying and demonstrating efficacy marker in an animal model(s) applicable to predicting protective immunity in humans, and demonstrating preliminary safety and efficacy against an aerosol challenge in a relevant animal model. Conduct GLP safety and toxicity studies in animal model systems. Identify endpoints of clinical efficacy or its surrogate in animal models that may be applicable to predicting protective immunity in humans. Conduct studies to evaluate immunogenicity, as well as pharmacokinetics and pharmacodynamics when appropriate. Stability studies initiated. | Further development. Devices compared to existing modalities and indications for use and equivalency demonstrated in model systems. Examples include devices tested through simulation, in tissue or organ models, or animal models if required. |
| 6 | Phase 1 clinical trials are conducted to demonstrate safety of candidates in a small number of subjects under carefully controlled and intensely monitored clinical conditions. Evaluation of immunogenicity and/or pharmacokinetics and pharmacodynamics data to support design of Phase 2 clinical trials. Surrogate efficacy models are validated. | Clinical trials are conducted to demonstrate safety of candidate Class III medical device in a small number of humans under carefully controlled and intensely monitored clinical conditions. Production technology demonstrated through production-scale cGMP plant qualification. |
| 7 | Phase 2 safety and immunogenicity trials are conducted. Product immunogenicity and biological activity (e.g., preliminary evidence of efficacy) are determined. Product final dose, dose range, schedule, and route of administration are established from vaccine immunogenicity and biologic activity and, when necessary, from clinical pharmacokinetics and pharmacodynamics data. Phase 2 clinical trials completed. | Clinical safety and effectiveness trials are conducted with a fully integrated Class III medical device prototype in an operational environment. Continuation of closely controlled studies of effectiveness and determination of short-term adverse events and risks associated with the candidate product. Functional testing of candidate devices is completed and confirmed, resulting in final downselection of prototype device. Clinical safety and effectiveness trials are completed. Final product design is validated, and final prototype and/or initial commercial scale device is produced. |
| 8 | Implementation of expanded Phase 3 clinical trials or surrogate tests to gather information relative to the safety and effectiveness of the candidate biologic/vaccine. Trials are conducted to evaluate the overall risk-benefit of administering the candidate product and to provide an adequate basis for product labelling. Process validation is completed and followed by lot consistency/reproducibility studies. | Implementation of clinical trials to gather information relative to the safety and effectiveness of the device. Trials are conducted to evaluate the overall risk-benefit of using the device and to provide an adequate basis for product labelling. |
| 9 | The pharmaceutical (i.e., biologic or vaccine) or medical device can be distributed/marketed. Post-marketing studies (non-clinical or clinical) may be required. Post-marketing surveillance. | The medical device can be distributed/ marketed. Post-marketing studies (non-clinical or clinical) may be required. Post-marketing surveillance. |
| Table S4: A more detailed description of TRLs for biological pharmaceuticals and medical devices. This table has been adapted from the US Department of Defense Technology Readiness Assessment Deskbook (Office of the director of defense and engineering, 2009), with some modifications to the text. Note that the various gene therapies used in the reviewed studies could be classified as either biologics or medical devices based on their use of scaffolds, hence both TRA frameworks are presented here.  **Wordcloud ignore word list for automated publication analysis:** bone_formation, bone_defect, large_animal, critical_size_defect, growth_factor, control_group, dental_implant, large_animal_model, gene_transfer_bone, large_animal_model, compared_control, gene_therapy, gene_transfer, compared_control, group_compared, bone_formation_month_hematoxylin_eosin, protein_bmp, bone_healing, autologous_bone, animal_model, gene_delivery, critical_size, solid_bone_formation_month_hematoxylin, compared_group, human_bone, solid_bone_formation_month, analysis_demonstrated_difference_bone, tissue_engineering, human_bmp, bone_formation_month_hematoxylin, human_bone_morphogenetic_protein, bone_morphogenetic, bonre_regeneration, repair_orbital_wall_defect, formation_month_hematoxylin_eosin, morphogenetic_protein, bone_defect_compared, group_compared_group, computed_tomography, compared_control_group, group_week, vivo_gene_therapy, defect_compared, higher_bone_formation, compared_group_week | | |
